# A Bayesian Approach to Understanding Sex Differences in Tuberculosis Disease Burden

**DOI:** 10.1093/aje/kwy131

**Published:** 2018-06-27

**Authors:** Katherine C Horton, Tom Sumner, Rein M G J Houben, Elizabeth L Corbett, Richard G White

**Affiliations:** 1 Department of Clinical Research, London School of Hygiene and Tropical Medicine, London, United Kingdom; 2 Tuberculosis Modelling Group, Tuberculosis Centre, London School of Hygiene and Tropical Medicine, London, United Kingdom; 3 Department of Infectious Disease Epidemiology, London School of Hygiene and Tropical Medicine, London, United Kingdom; 4 Malawi-Liverpool-Wellcome Trust Clinical Research Programme, Blantyre, Malawi

**Keywords:** access to health care, Bayes theorem, gender, incidence, mathematical model, sex, time to treatment, tuberculosis

## Abstract

Globally, men have a higher epidemiologic burden of tuberculosis (incidence, prevalence, mortality) than women do, possibly due to differences in disease incidence, treatment initiation, self-cure, and/or untreated-tuberculosis mortality rates. Using a simple, sex-stratified compartmental model, we employed a Bayesian approach to explore which factors most likely explain men’s higher burden. We applied the model to smear-positive pulmonary tuberculosis in Vietnam (2006–2007) and Malawi (2013–2014). Posterior estimates were consistent with sex-specific prevalence and notifications in both countries. Results supported higher incidence in men and showed that both sexes faced longer durations of untreated disease than estimated by self-reports. Prior untreated disease durations were revised upward 8- to 24-fold, to 2.2 (95% credible interval: 1.7, 2.9) years for men in Vietnam and 2.8 (1.8, 4.1) years for men in Malawi, approximately a year longer than for women in each country. Results imply that substantial sex differences in tuberculosis burden are almost solely attributable to men’s disadvantages in disease incidence and untreated disease duration. The latter, for which self-reports provide a poor proxy, implies inadequate coverage of case-finding strategies. These results highlight an urgent need for better understanding of gender-related barriers faced by men and support the systematic targeting of men for screening.

Substantial sex disparity exists in the burden of tuberculosis, as indicated by incidence, prevalence, and mortality estimates. Each year, more tuberculosis cases are reported among men than among women globally and in most countries (1). Prevalence surveys, which provide the most reliable source of data on tuberculosis burden (2), show even greater sex disparity, with a 2-fold higher underlying burden of undiagnosed disease among men than among women in low- and middle-income countries (3). Comparisons of these 2 measures using prevalence-to-notification ratios (4) imply that gaps in the detection and reporting of new cases are greater for men than for women (3, 5). Yet discussions of gender and tuberculosis tend to focus on and prioritize the needs of women (6–9), often highlighting women as a key population with need for improved access to tuberculosis services (6–8).

Sex disparities in the epidemiologic burden of tuberculosis could be explained by sex differences in 4 factors: disease incidence, treatment initiation, self-cure, and/or untreated-tuberculosis mortality rates. Individuals are added to the pool of prevalent cases upon development of incident disease, and disease incidence rates could differ between men and women due to sex differences in exposure to infection and/or susceptibility to disease. Diseased individuals are then removed from the prevalent pool by successfully initiating treatment, naturally clearing themselves of disease (“self-cure”), or dying (10), rates of which may differ according to sex due to biological and/or sociobehavioral factors. Existing evidence suggests that there may be sex differences in disease incidence and treatment initiation rates, while there is no evidence to support differences in self-cure or untreated-tuberculosis mortality rates (11).

Using a simple compartmental model (12) of tuberculosis incidence, prevalence, and case notification rates, we employed a Bayesian approach to explore which factors most likely explain the higher epidemiologic burden of disease in men. A better understanding of sex differences in the burden of tuberculosis is imperative for the formulation of evidence-based gender-sensitive policies and programs. Implementing such programs at both the global and national level will improve equity for the sexes in access to diagnosis and treatment, as prioritized in the End Tuberculosis Strategy (13) and Sustainable Development Goals (14). Although little attention is placed on men’s burden of disease in current gender-sensitive policies and programs (6–9), addressing sex imbalances in tuberculosis will ultimately benefit men, women, and children.

## METHODS

### Data

We performed analyses for smear-positive pulmonary tuberculosis in 2 settings: Vietnam, where the male-to-female ratio in smear-positive tuberculosis prevalence is one of the highest in the world at 5.1:1 (15), and Malawi, where the corresponding sex disparity is less extreme, with a male-to-female prevalence ratio of 2.0:1 (16).

Sex-specific tuberculosis incidence rates were based on 2015 World Health Organization (WHO) estimates of incident cases (age ≥15 years) (17) and population estimates from the United Nations Department of Economic and Social Affairs (18, 19). Treatment initiation rates for men and women were calculated as the inverse of untreated disease duration based on self-reported time from disease onset to treatment initiation (20, 21), as extracted from literature reviews (Web Appendix 1, Web Tables 1–3). Self-cure and untreated-tuberculosis-specific mortality rates were gathered from sources used in previous modeling studies (22–24). Untreated-tuberculosis-specific mortality and background mortality (25) rates were combined to give an overall untreated-tuberculosis mortality rate. Log-normal distributions, which provided the best fit to the data, were used to describe disease incidence, treatment initiation, self-cure, and untreated-tuberculosis mortality rates. Informative priors were chosen so that the middle 95% of expected values fell within the 95% confidence interval, or the middle 50% of expected values fell within the interquartile range, as appropriate to available data. Distributions were fitted using Parameter Solver, version 3.0, a software application that solves for the distribution parameters of a random variable given user-defined quantiles (26).

Sex-specific prevalence data for smear-positive tuberculosis were collated from national prevalence surveys conducted in Vietnam in 2006–2007 (15) and in Malawi in 2013–2014 (16). Case notification rates for smear-positive tuberculosis in men and women were calculated using case notification numbers reported to WHO (1) and population estimates from the United Nations Department of Economic and Social Affairs (18, 19). Confidence intervals for prevalence and case notification rates were based on the normal approximation to the binomial distribution.

**Figure 1.**
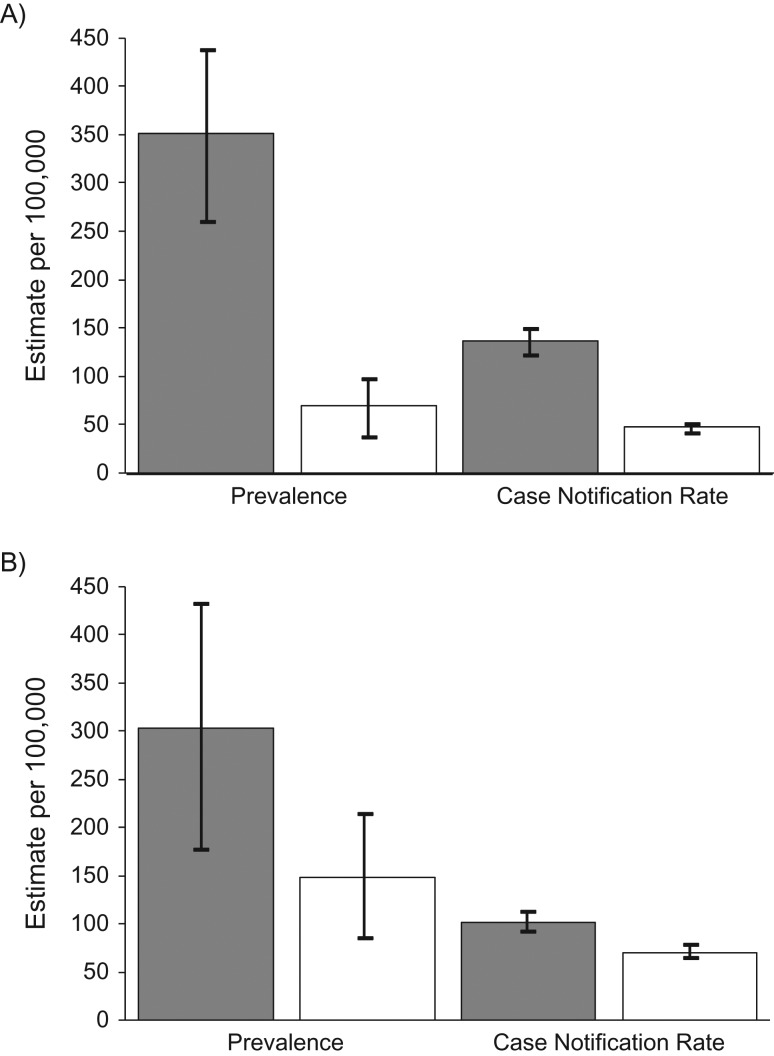
Prevalence and case notification rates for tuberculosis, according to sex, in Vietnam (2006–2007) (A) and Malawi (2013–2014) (B). Male-to-female ratios in prevalence-to-notification ratios: 1.75 (95% credible interval: 1.21, 2.58) in Vietnam and 1.41 (95% credible interval: 0.91, 2.20) in Malawi. Dark gray bars indicate distributions for men, white bars indicate distributions for women, and lines indicate 95% confidence intervals.

Full details of prior specification and data are provided in the Web Appendices 1 and 2 and Web Tables 4–6, and data on prevalence and case notification rates are shown in Figure 1.

### Model

**Figure 2.**
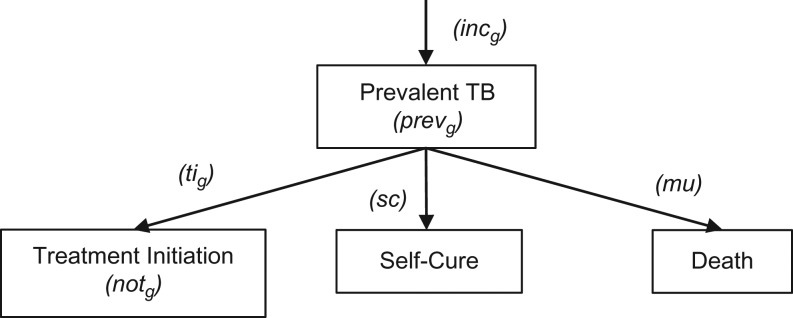
A sex-stratified (male and female) model of disease incidence, prevalence, and case notification rates for adult (age ≥15 years) smear-positive tuberculosis. In this model, *prev*_*g*_ is the tuberculosis prevalence in sex *g*, *not*_*g*_ is the case notification rate in sex *g*, *inc*_*g*_ is the disease incidence rate in sex *g*, *ti*_*g*_ is the treatment initiation rate (inverse of untreated disease duration) in sex *g*, *sc* is the self-cure rate, and *mu* is the untreated-tuberculosis mortality rate.

We developed a simple sex-stratified (male and female) model of disease incidence, prevalence, and case notification rates for adult (age ≥15 years) smear-positive tuberculosis, as shown in Figure 2. (Direct acyclic graphs are provided in Web Figures 1 and 2 in Web Appendix 3.) Transition rates for disease incidence, treatment initiation, self-cure, and untreated-tuberculosis mortality were used to calculate expected prevalence and case notification rates. Sex-related risk factors for infection with *Mycobacterium tuberculosis*, progression to tuberculosis disease, and death following infection—notably tobacco smoking in Vietnam and human immunodeficiency virus in Malawi—were not explicitly modeled, instead being captured as part of overall sex differences in disease incidence and untreated-tuberculosis mortality rates.

The expected prevalence of smear-positive tuberculosis in sex *g*, *prev*_*g*_, was calculated as *prev*_*g*_*= inc*_*g*_**÷***(ti*_*g*_*+ sc + mu)* where *inc*_*g*_ is the disease incidence rate in sex *g*, *ti*_*g*_ is the treatment initiation rate in sex *g*, *sc* is the self-cure rate, and *mu* is the untreated-tuberculosis mortality rate. The expected case notification rate for smear-positive tuberculosis in sex *g*, *not*_*g*_, was calculated as *not*_*g*_*= prev*_*g*_*× ti*_*g*_. Male-to-female ratios in expected prevalence-to-notification ratios were calculated.

### Statistical analysis

The evidence described above was used to specify model priors for disease incidence, treatment initiation, self-cure, and untreated-tuberculosis mortality for each sex in each country (Tables 1 and 2, “Model Priors”). The model was then confronted with sex-specific data on prevalence and case notification rates (Tables 1 and 2, “Empirical Data”) in a Bayesian framework (27). Posterior model estimates (Tables 1 and 2, “Model Posteriors”) show how prior beliefs about disease incidence, treatment initiation, self-cure, and untreated-tuberculosis mortality should be modified in light of the empirical prevalence and notification data. Posterior estimates were examined for consistency with empirical data on sex-specific prevalence and case notification rate, as well as male-to-female ratios in prevalence-to-notification ratios. The model was considered consistent with empirical data if empirical point estimates were within the 95% credible intervals of posterior model estimates.

**Table 1.** Model Priors, Empirical Data, and Model Posteriors for a Bayesian Analysis of Sex Disparities in the Epidemiological Burden of Tuberculosis, Vietnam, 2006–2007

Parameter	Model Priors^a^		Empirical Data		Model Posteriors^a^	
	Median	95% CrI	Estimate	95% CI	Median	95% CrI
Incidence rate, annual per 100,000^b^						
Male	245	192, 312			258	216, 314
Female	58	27, 124			67	56, 83
Untreated disease duration, years^c^						
Male	0.09	0.02, 0.39			2.20	1.65, 2.89
Female	0.11	0.03, 0.38			1.01	0.60, 1.59
Self-cure rate, annual						
Male and female	0.19	0.09, 0.41			0.17	0.09, 0.31
Untreated-TB mortality rate, annual						
Male and female	0.29	0.11, 0.77			0.21	0.10, 0.41
Prevalence, per 100,000^b^						
Male			351	262, 440	305	234, 389
Female			69	39, 99	48	29, 75
Notification, per 100,000^b^						
Male			137	123, 151	138	125, 153
Female			47	42, 52	48	43, 53
Prevalence-to-notification ratio						
Male-to-female ratio			1.75	1.21, 2.58	2.18	1.28, 3.90

Abbreviations: CI, confidence interval; CrI, credible interval; TB, tuberculosis.

^a^ All potential scale reduction factors, which equal 1 at convergence, were between 1.001 and 1.003.

^b^ Modeled as proportion but shown as number per 100,000 population.

^c^ Untreated disease duration is the inverse of treatment initiation rate.

**Table 2.** Model Priors, Empirical Data, and Model Posteriors for a Bayesian Analysis of Sex Disparities in the Epidemiological Burden of Tuberculosis, Malawi, 2013–2014

Parameter	Model Priors^a^		Empirical Data		Model Posteriors^a^	
	Median	95% CrI	Estimate	95% CI	Median	95% CrI
Incidence rate, annual per 100,000^b^						
Male	354	235, 534			295	218, 410
Female	151	57, 402			161	118, 235
Untreated disease duration, years^c^						
Male	0.22	0.07, 1.03			2.77	1.83, 4.06
Female	0.23	0.06, 1.86			1.88	1.17, 2.86
Self-cure rate, annual						
Male and female	0.19	0.09, 0.41			0.22	0.10, 0.47
Untreated-TB mortality rate, annual						
Male and female	0.30	0.12, 0.78			0.43	0.18, 0.87
Prevalence, per 100,000^b^						
Male			303	176, 431	286	191, 413
Female			149	85, 213	134	84, 201
Notification, per 100,000^b^						
Male			102	91, 112	103	93, 113
Female			71	64, 78	71	65, 78
Prevalence-to-notification ratio						
Male-to-female ratio			1.42	0.91, 2.20	1.48	0.83, 2.73

Abbreviations: CI, confidence interval; CrI, credible interval; TB, tuberculosis.

^a^ All potential scale reduction factors, which equal 1 at convergence, were between 1.001 and 1.003.

^b^ Modeled as proportion but shown as number per 100,000 population.

^c^ Untreated disease duration is the inverse of treatment initiation rate.

Posterior model estimates were calculated using Markov chain Monte Carlo algorithm in WinBUGS (28) via R (R Foundation for Statistical Computing, Vienna, Austria) (29) according to code included in Web Appendix 4. Results were based on 3 Markov chains of 21,000 iterations; the first 1,000 samples of each chain were discarded as burn-in. Convergence was assessed visually and using potential scale reduction factors (30).

### Sensitivity analyses

We conducted extensive sensitivity analyses, which are described in detail in Web Appendices 5–7. We explored our choice of model structure by examining all combinations of fixing individual parameters by sex and allowing individual parameters to differ according to sex. We also explored our choice of incidence rate priors using incidence rate priors based on estimates from the Institute for Health Metrics and Evaluation (IHME) (31). Finally, we explored the implications of assuming self-reports of symptom duration prior to treatment accurately describe untreated disease duration.

## RESULTS

Prior model estimates accurately represented evidence on disease incidence, treatment initiation, self-cure rate, and untreated-tuberculosis mortality rates.

Posterior model estimates were consistent with empirical data on sex-specific prevalence and case notification rates, as well as male-to-female ratios in prevalence-to-notification ratios, in both countries (Tables 1 and 2).

In both countries, posterior incidence rate estimates were consistent with empirical data. In Vietnam, incidence was estimated as (posterior median) 258 (95% credible interval, CrI: 216, 314) per 100,000 men and 67 (95% CrI: 56, 83) per 100,000 women. In Malawi, incidence was estimated as (posterior median) 295 (95% CrI: 218, 410) per 100,000 men and 161 (95% CrI: 118, 235) per 100,000 women.

**Figure 3.**
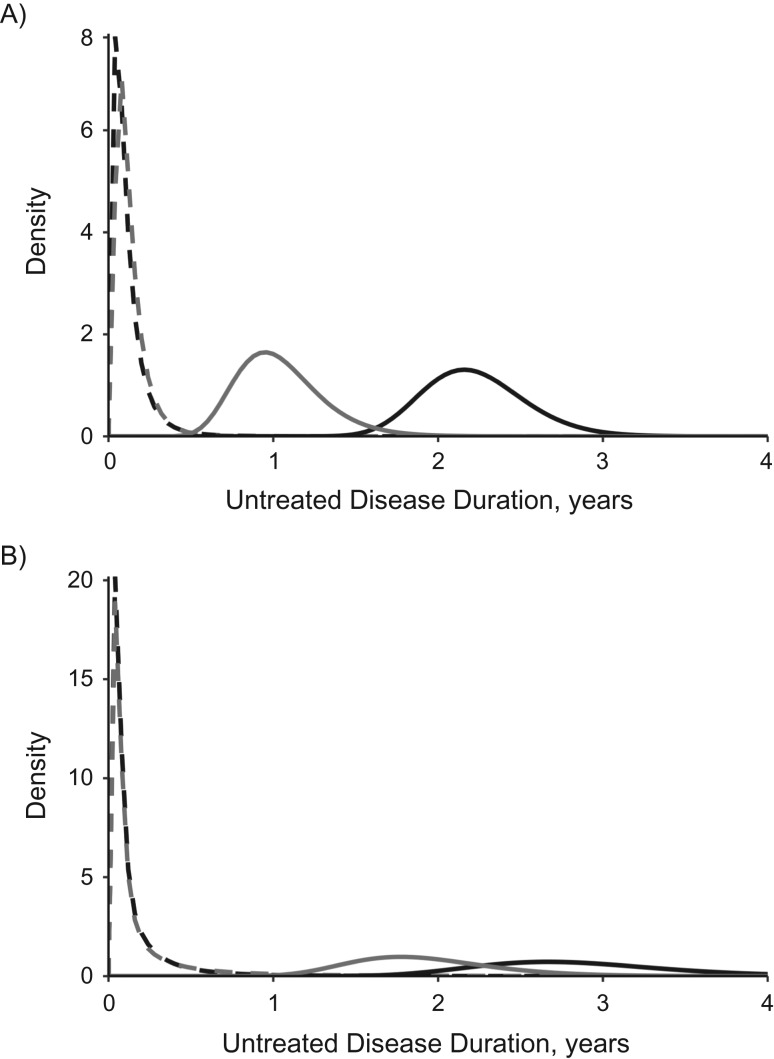
Density plots for prior and posterior distributions for untreated tuberculosis disease duration, according to sex, in Vietnam (2006–2007) (A) and Malawi (2013–2014) (B). Prior distributions are shown as dashed lines, and posterior distributions are shown as solid lines. Dark gray lines indicate distributions for men; light gray lines indicate distributions for women.

Posterior estimates for treatment initiation rate showed that both men and women faced a much longer time between onset of disease and initiation of treatment than estimated from self-reports of symptom duration prior to treatment (Figure 3). Prior untreated disease durations were revised upward 8- to 24-fold, to posterior median estimates of 2.2 (95% CrI: 1.7, 2.9) and 2.8 (95% CrI: 1.8, 4.1) years for men in Vietnam and Malawi, respectively, and 1.0 (95% CrI: 0.6, 1.6) and 1.9 (95% CrI: 1.2, 2.9) years for women, respectively.

In both countries, sensitivity analyses around model structure (Web Table 7) supported our decision to allow only disease incidence and treatment initiation rates to differ according to sex (Web Tables 8–22). In these analyses, all scenarios that allowed rates of disease incidence and treatment initiation to differ according to sex (regardless of restrictions on self-cure and untreated-tuberculosis mortality rates) were consistent with empirical data (Web Tables 18, 19, 22). When self-cure and/or untreated-tuberculosis mortality rates, in addition to disease incidence and treatment initiation rates, were allowed to differ according to sex, posterior estimates were not substantially different from the main analysis (Web Tables 18, 19, 22). In Malawi, posterior estimates from 2 additional scenarios were also consistent with empirical data (Web Tables 16 and 21). However posterior estimates for untreated-tuberculosis mortality rates among women in these scenarios were over twice those estimated among men, which evidence suggests is unlikely (11). No other scenarios produced posterior estimates consistent with empirical data.

Sensitivity analyses were also conducted using incidence estimates from IHME rather than WHO (Web Table 23). Posterior estimates for Vietnam were not consistent with empirical data on sex-specific prevalence or male-to-female ratios in prevalence-to-notification ratios. This is likely a result of IHME underestimating sex disparity in disease incidence in Vietnam. While WHO estimates that incidence is over 4 times higher in men than in women, IHME estimates that incidence in men is only 50% higher than in women (Web Figure 3). In contrast, in Malawi, where both WHO and IHME estimate that incidence among men is approximately twice that among women (Web Figure 4), posterior estimates were consistent with empirical data on sex-specific prevalence and case notification rates, as well as male-to-female ratios in prevalence-to-notification ratios (Web Table 24).

Final sensitivity analyses assumed self-reports of symptom duration prior to treatment accurately described untreated disease duration and examined the impact of this assumption on disease burden estimates. Posterior prevalence estimates from these analyses were only 4% to 12% of those reported in recent prevalence surveys (for example, 13 (95% CrI: 12, 15) per 100,000 men in Vietnam) and male-to-female ratios in prevalence-to-notification ratios significantly less than 1 (Web Table 25). These results illustrate how unlikely it is that self-reports of symptom duration are accurate measures of untreated disease duration in light of recent prevalence surveys.

## DISCUSSION

Our results imply that the substantial sex differences in the epidemiologic burden of tuberculosis are almost solely attributable to sex differences in disease incidence and treatment initiation rates, both of which disadvantage men. Although differences between self-reported symptom duration prior to treatment and our model posteriors indicate that both men and women face long periods of undiagnosed tuberculosis disease prior to treatment, men face substantially longer durations of untreated disease. Improved access to tuberculosis diagnostic and treatment services is needed for all individuals, but with more pressing need to better understand and address men’s barriers to care.

Our model confirms sex differences in tuberculosis incidence that have already been acknowledged to some extent in estimates from WHO (17) and IHME (31). Men’s higher incidence of disease may be a result of a number of factors, including biological susceptibility (32, 33), social contact patterns (34, 35), tobacco smoking (36), alcohol consumption (37), and/or undiagnosed or untreated human immunodeficiency virus infection (38, 39). While the relative contribution of these different factors is not well-understood, it is clear that there are more new cases of tuberculosis among men than among women in both Vietnam and Malawi, and likely in other countries where similar sex differences in prevalence are found (3).

We also found that prevalence and case notification data are simply not consistent with a longer untreated disease duration in women than in men, despite the widespread recognition of women as a key population with need for improved access to tuberculosis services (6–8). Our results imply that men either have lower symptom awareness or face greater barriers in accessing tuberculosis care than women. Men tend to present with more advanced disease and show lower health-care utilization for tuberculosis (40), as for many infectious and noninfectious conditions (38, 39, 41, 42). Men’s health-care decisions are rooted in societal constructs of masculinity, including concepts that lead to societal pressure to neglect symptoms in order to be physically strong and to fulfill roles as the leader and provider for their immediate and extended family (43–48). Although women with tuberculosis may face greater delays in receiving appropriate medical attention after seeking care (40), our findings suggest that, on average, men’s delays in seeking health care far outweigh any delays women face after seeking care in these 2 countries.

Timely access to tuberculosis care is essential for successful patient outcomes and for the prevention of transmission, yet current evidence points to considerable delays between the onset of disease and treatment initiation (49–52). Our findings urge caution in the interpretation of self-reports as a measure of untreated disease duration; these estimates appear to substantially underestimate time to treatment initiation for both sexes. Patients usually report the time from symptom onset to treatment initiation in terms of weeks (49–52), yet our results and those of others (4, 11, 24, 49–54) suggest instead that years pass between the development of disease and treatment initiation. Self-reports may be limited by recall accuracy and different perceptions of disease. They may also fail to capture the full duration of long-term illness characterized by remissions and relapses along a continuum (55) rather than unrelenting progression of symptom severity (10, 55, 56). It is likely that patients report only the duration of the most recent episode of “acute-on-chronic” symptom deterioration that has led directly to care-seeking and diagnosis, rather than the full duration of untreated disease. The marked differences between self-reported time to treatment initiation and our posterior estimates may explain in part why current passive case-finding strategies have not been as successful as initially projected, despite global implementation (57).

There are several limitations to the results presented here. The model chosen for this analysis was deliberately simple in order to clearly define and examine the overall impact of each parameter within the model. As such, results provided here describe median untreated disease duration and do not take into account heterogeneity within the populations of interest. Furthermore, we cannot assess the relative contribution of specific biological and sociocultural factors to increased disease incidence and untreated disease duration among men. In addition, we have not included any consideration of smear-negative and extrapulmonary tuberculosis disease, for which untreated disease duration is likely even longer than described here, although it seems unlikely that the sex differences found here would disappear when other disease types are considered.

Our results imply that the substantial sex differences in the epidemiologic burden of tuberculosis are almost solely attributable to sex differences in disease incidence and treatment initiation rates, both of which disadvantage men. Our results add weight to the growing body of evidence that men have a higher incidence of tuberculosis disease (17, 31) and also often face longer delays than women in accessing treatment (3–5). Self-reported symptom duration prior to treatment provides a poor proxy for untreated disease duration for both sexes, especially for men. In both Vietnam and Malawi our posterior median estimates suggest that men spent over a year longer than women prior to initiating treatment for tuberculosis disease.

Despite male disadvantage in accessing care and strong evidence that men have a higher epidemiologic burden of disease, discussions of gender and tuberculosis tend to focus on and prioritize the needs of women. There is little consideration that men face substantial gender-related barriers of their own when accessing tuberculosis diagnosis and treatment. National and international tuberculosis programs need to reconsider gender disparity as a barrier to achieving the ambitious elimination goals set for tuberculosis under the End Tuberculosis Strategy (13) and the Sustainable Development Goals (14), from the perspective of men.

Acknowledging men as a disadvantaged group with limited access to timely diagnosis and treatment is only a first step. The long duration of untreated disease estimated here, particularly among men, implies inadequate coverage of current case-finding strategies. Action is needed to ensure that men are not being unduly disadvantaged by the prominent focus on maternal and child health that characterizes primary care in many countries. Steps must be taken to acknowledge and address the ways in which constructions of masculinity add to and interact with health system barriers that affect men’s health-seeking behaviors. Systematic screening offers an opportunity to expedite diagnosis with less reliance on severe symptoms (58). The consideration of screening programs predominately aimed at men is supported by our data showing them to be a high-prevalence, high-incidence subgroup with longer untreated disease duration than women.

## Supplementary Material

Web Material

## Abbreviations

CrI: credible interval
IHME: Institute for Health Metrics and Evaluation
WHO: World Health Organization
