# Supplementary material for: A Bayesian Approach to Understanding Sex Differences in Tuberculosis Disease Burden
Source: Am J Epidemiol. 2018 Jun 27;187(11):2431–8. doi: 10.1093/aje/kwy131 (PMC6211250; doi:10.1093/aje/kwy131)
Supplement: Web Material [file kwy131_horton_web_material_final.pdf]

Web Material

A Bayesian Approach To Understanding Gender Differences In Tuberculosis Disease Burden

Katherine C. Horton, Tom Sumner, Rein M.G.J. Houben,
Elizabeth L. Corbett, and Richard G. White

Web Appendix 1: Priors **2**
Web Appendix 2: Data **9**
Web Appendix 3: Direct Acyclic Graphs **11**
Web Appendix 4: Code **12**
Web Appendix 5: Sensitivity Analyses – Model Structure **18**
Web Appendix 6: Sensitivity Analyses – Incidence Rate **35**
Web Appendix 7: Sensitivity Analyses – Treatment Initiation Rate **39**
Web References **41**

WEB APPENDIX 1: PRIORS

All priors, model estimates and data on which priors are based are summarised at the end of this section in Web Table 4 and Web Table 5.

DISEASE INCIDENCE RATE

Disease incidence estimates were calculated from World Health Organization (WHO) tuberculosis (TB) country profile estimates of the number of incident cases in men and women ≥ 15 years of age in 2015 (1) combined with 2015 national population estimate for individuals ≥ 15 years of age from the United Nations World Population Prospect (2, 3). Although we would have preferred to match the year of disease incidence estimates to that of other data sources (e.g., prevalence, case notification rate), 2015 was the only year for which gender-specific incidence estimates were available from WHO. Similarly, only overall disease incidence estimates for all forms of TB were available, rather than estimates for smear-positive TB alone.

Overall TB disease estimates are derived from prevalence survey results, combined with estimates of disease duration. To calculate gender-specific disease incidence, WHO applies the male-to-female (M:F) ratio in case notifications to overall disease incidence estimates, assuming no gender differential in detection of incident cases and acknowledging that the proportion of disease incidence among men is likely underestimated due to this assumption (4).

In Viet Nam, there were an estimated 88,000 (95% CI 67,000-109,000) incident cases of TB among men and 28,000 (95% CI 10,000-46,000) incident cases among women in 2015 (1). Given 2015 population estimates of 34,964,611 men and 36,906,231 women (2, 3), the corresponding disease incidence rate is estimated at 252 (95% CI 192-312) per 100,000 men and 76 (95% CI 27-125) per 100,000 women. Overall estimates suggest there were 116,000 (95% CI 101,000-131,000) total incident cases of TB in 2015 (1), which corresponds to an overall disease incidence rate of 161 (95% CI 141-182) per 100,000 population.

In Malawi, there were an estimated 18,000 (95% CI 11,000-25,000) incident cases of TB among men and 11,000 (95% CI 2,700-19,000) incident cases among women in 2015 (1). Given population estimates of 4,679,650 men and 4,761,703 women (2, 3), the corresponding disease incidence rate is estimated at 385 (95% CI 235-534) per 100,000 men and 231 (95% CI 57-399) per 100,000 women. Overall estimates suggest there were 29,000 (95% CI 19,000-39,000) total incident cases of TB in 2015 (1), which corresponds to an overall disease incidence rate of 307 (95% CI 201-413) per 100,000 population.

Disease incidence estimates were fitted to log-normal distributions with the middle 95% of probabilities falling within the 95% confidence interval.

TREATMENT INITIATION RATE

A literature review was conducted to identify studies describing the progression of smear-positive TB patients through the TB care pathway. Studies conducted in Viet Nam or Malawi were identified through searches of PubMed, Embase, Global Health and the Cochrane Library using standardised search terms (Web Table 1). Searches were last updated 26 April 2017.

Web Table 1: Database search terms

Database	Country	
	Viet Nam	Malawi
PubMed	viet* AND ("chronic cough" OR tubercul*) AND (seek* OR access* OR utilis* OR utiliz* OR delay* OR los* OR default* OR adher* OR complet* OR outcome)	malawi* AND ("chronic cough" OR tubercul*) AND (seek* OR access* OR utilis* OR utiliz* OR delay* OR los* OR default* OR adher* OR complet* OR outcome)
Embase/ Global Health	(viet* and ("chronic cough" or tubercul*) and (seek* or access* or utilis* or utiliz* or delay* or los* or default* or adher* or complet* or outcome)).af	(malawi* and ("chronic cough" or tubercul*) and (seek* or access* or utilis* or utiliz* or delay* or los* or default* or adher* or complet* or outcome)).af
Cochrane	(viet* and ("chronic cough" or tubercul*) and (seek* or access* or utilis* or utiliz* or delay* or los* or default* or adher* or complet* or outcome)) in Title, Abstract, Keywords in Trials	(malawi* and ("chronic cough" or tubercul*) and (seek* or access* or utilis* or utiliz* or delay* or los* or default* or adher* or complet* or outcome)) in Title, Abstract, Keywords in Trials

For Viet Nam, 377 records were screened by title and abstract; 19 full-text articles were assessed for eligibility; 10 relevant studies were identified, of which four reported results by gender (5-8).

For Malawi, 452 records were screened by title and abstract; 48 full-text articles were assessed for eligibility; 18 relevant studies were identified, of which five reported results by gender (9-13).

Studies reporting results by gender were evaluated for their ability to form a complete path from onset of symptoms to treatment initiation (alone or in combination with data from another study) and whether reported results included estimates of uncertainty in addition to point estimates. Selected studies are described below. Studies reporting results by gender but not selected for inclusion are described in Web Table 2.

Web Table 2: Studies reporting results by gender but not included in final prior estimates

Viet Nam			
Study description	Results	Reason for exclusion	Reference
National survey of 4381 TB suspects (2006-07)	Mean time from onset of cough to first attendance at a health care facility was 4.4 weeks (95% CI 4.1-4.7) for men and 3.6 (95% CI 3.3-4.0) weeks for women.	Cannot be combined with another data source to form complete path from onset to treatment initiation.	Hoa 2011 (5)
Outpatient survey of 1027 TB patients in 23 districts in four provinces (1996)	Mean time from symptom onset to TB diagnosis was 13.3 weeks (95% CI 11.5-15.1) for women and 11.4 weeks (95% CI 10.6-12.2) for men.	Cannot be combined with another data source to form complete path from onset to treatment initiation.	Long 1999 (6)
Community-based survey of 492 chronic coughers in Ha Tay province (pre-	Mean time from onset of symptoms to hospital treatment was longer for women (41 days)	Cannot be combined with another data source to form complete path from onset	Thorson 2000 (7)

2000)	than men (19 days).	to treatment initiation.	
Malawi			
Study description	Results	Reason for exclusion	Reference
Outpatient survey of 290 men and 257 women with TB in six districts in three regions (pre-2006)	Mean time from symptom onset to sputum examination was 58.1 days for men and 64.6 days for women. No difference between men and women.	No estimate of uncertainty around mean reported.	Weiss 2006 (9)
Appx. 100 TB patients in Lilongwe (pre-2008)	58% of men and 42% of women diagnosed within 30 days of symptom onset; 18% of men and 30% of women diagnosed after delay of more than 90 days.	Cannot be combined with another data source to form complete path from onset to treatment initiation.	Gosoniu 2008 (10)
Interview of 598 TB patients in Karonga district (1996-2001)	Median duration from onset of cough to treatment initiation was appx. 2 months. No difference between men and women.	Results shown graphically but not reported numerically for men and women.	Crampin 2004 (11)
Comparison of programme registers in rural Ntcheu district (2000)	Appx. 15% of men and women lost to follow-up between diagnosis and treatment initiation.	Results report proportion progressing through care pathway rather than duration of progression.	Squire 2005 (12)

Appx.: approximately; CI: confidence interval

In Viet Nam, a cross-sectional survey of consecutively-enrolled new TB patients treated by the National TB Control Programme in 70 randomly selected districts in one quarter of 2002 stated that men (n=1491) reported a median of 4 (inter-quartile range, IQR, 3-8) weeks interval from cough onset to treatment initiation, while women (n=596) reported a median of 5 (IQR 4-9) weeks interval (8). Among all study participants (n=2093), the median reported time from cough onset to treatment initiation was 4 (IQR 3-8) weeks (8).

In Malawi, a cross-sectional survey of 588 pulmonary TB patients conducted in three TB centres in Blantyre, Lilongwe, and Mzuzu between July and December 2011 stated that men (n=304) reported a median of 14 (IQR 14-28) days from onset of TB symptoms to visiting any healthcare provider; men then reported a median of 59 (IQR 28-99) days from visiting any healthcare provider to initiating treatment (13). Women (n=156) reported a median of 21 (IQR 14-30) days from onset of TB symptoms to visiting any healthcare provider and a median of 64 (IQR 24-125) days from visiting any healthcare provider to initiating treatment (13). Among all study participants (n=460), the median reported time from onset of TB symptoms to visiting any healthcare provider was 14 (IQR 14-28) days, and the median reported time from visiting any healthcare provider to initiating treatment was 59 (IQR 27-108) days (13).

For each time interval of interest, data reported in days or weeks were converted to years (assuming 52 weeks or 365 days in a year) and fitted to a log-normal distribution with the middle 50% of probabilities falling within the IQR.

In Malawi, log-normal distributions for estimates of time from onset of TB symptoms to visiting any healthcare provider and time from visiting any healthcare provider to initiating treatment were summed to

give a log-normal distribution of time from onset of TB symptoms to treatment initiation. The derivation of priors for self-reported symptom duration prior to treatment in Malawi is shown in Web Table 3.

Web Table 3: Derivation of priors for self-reported symptom duration prior to treatment based on self-reports in Malawi

Gender	A. Time from symptom onset to visiting any healthcare provider	B. Time from visiting any care provider to treatment initiation	C. Time from symptom onset to treatment initiation (A+B)
M & F	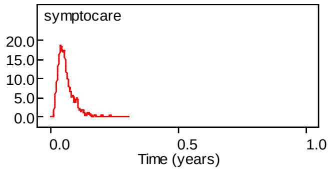	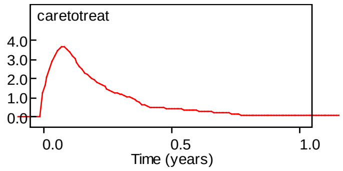	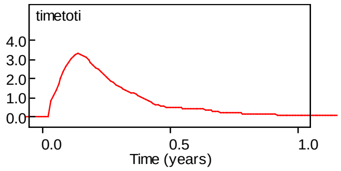
M	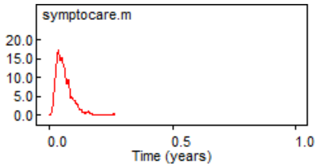	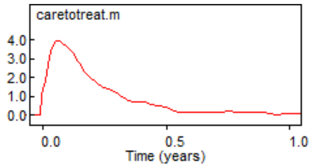	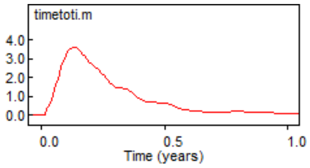
F	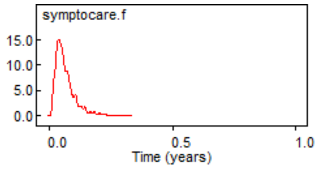	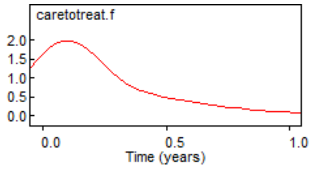	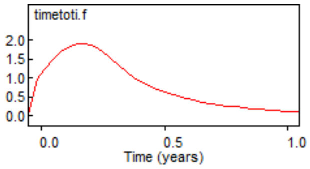

M: male; F: female

SELF-CURE RATE

The annual self-cure rate was taken from previous studies modelling TB transmission that have assumed this rate to be between 0.15 and 0.25 (15-17). A log-normal distribution was fitted to these data assuming the middle 50% of probabilities fall between 0.15 and 0.25. The range assumed by previous studies was treated as an IQR in fitting to reflect uncertainty around estimates for self-cure rate.

UNTREATED-TB MORTALITY RATE

Annual untreated-TB mortality rate estimates combine background mortality in the population and excess untreated-TB mortality attributable to smear-positive TB.

Background mortality was based on 2015 WHO estimates of life expectancy at birth, 71.3 years for men (annual rate of 0.014) and 80.7 years for women (annual rate of 0.012) in Viet Nam and 59.9 years for men (annual rate of 0.017) and 56.7 years for women (annual rate of 0.018) in Malawi (18). Excess mortality for untreated smear-positive TB has been assumed between 0.20 and 0.40 in previous modelling studies (15-17).

A log-normal distribution was fitted to the sum of these rates assuming the middle 50% of probabilities between 0.21 and 0.41 for Viet Nam and the middle 50% of probabilities between 0.22 and 0.42 for

Malawi. Ranges were treated as IQRs in fitting to reflect uncertainty around estimates for excess untreated smear-positive TB mortality rate.

Web Table 4: Data, model estimates and prior distributions for Viet Nam

Viet Nam				
Parameter	Gender	Data estimate (95% CI or range or IQR)	Model median (95% CrI)	Distribution in WinBUGS
Incidence rate (annual per 100,000*)	M & F	161 (95% CI 141-182)	161 (95% CrI 141-183)	dlnorm(-6.4365422,235.8498345)
	M	252 (95% CI 192-312)	245 (95% CrI 192-312)	dlnorm(-6.0126762,65.1874099)
	F	76 (95% CI 27-125)	58 (95% CrI 27-125)	dlnorm(-7.4508502,6.5428704)
Untreated disease duration (years)	M & F	0.08 (IQR 0.06-0.15)	0.09 (95% CrI 0.02-0.40 IQR 0.05-0.15)	dlnorm(-2.4474648,1.6973627)
	M	0.08 (IQR 0.06-0.15)	0.09 (95% CrI 0.02-0.40 IQR 0.05-0.15)	dlnorm(-2.4474648,1.6973627)
	F	0.10 (IQR 0.08-0.17)	0.11 (95% CrI 0.03-0.38 IQR 0.07-0.16)	dlnorm(-2.2355818,2.4323685)
Self-cure rate (annual)	M & F	0.20 (range 0.15-0.25)	0.19 (95% CrI 0.09-0.41 IQR 0.15-0.26)	dlnorm(-1.6417072,6.973734)
Untreated-TB mortality rate (annual)	M & F	0.31 (range 0.21-0.41)	0.29 (95% CrI 0.11-0.77 IQR 0.20-0.39)	dlnorm(-1.2261229,4.065313436)

M: male; F: female; CI: confidence interval; CrI: credible interval; IQR: inter-quartile range; Untreated disease duration: inverse of treatment initiation rate
* Modelled as proportion but shown as number per 100,000 population

Web Table 5: Data, model estimates and prior distributions for Malawi

Malawi				
Parameter	Gender	Data<br>estimate (95% CI or range or IQR)	Model<br>median (95% CrI)	Distribution in WinBUGS
Incidence rate (annual per 100,000*)	M & F	307 (95% CI 201-413)	288 (95% CrI 200-410)	dlnorm(-5.8495492,29.6291428)
	M	385 (95% CI 235-534)	354 (95% CrI 234-533)	dlnorm(-5.6429348,22.8071247)
	F	231 (95% CI 57-399)	152 (95% CrI 57-401)	dlnorm(-6.4969191,4.0579866)
Untreated disease duration (years)	M & F	0.04 (IQR 0.04-0.08) +<br>0.16 (0.07-0.30)	0.22 (95% 0.06-1.25 IQR 0.14-0.37)	dlnorm(-2.914242,3.788399) +<br>dlnorm(-1.872126,0.937561)
	M	0.04 (IQR 0.04-0.08) +<br>0.16 (IQR 0.08-0.27)	0.22 (95% 0.07-1.03 IQR 0.15-0.36)	dlnorm(-2.914242,3.788399) +<br>dlnorm(-1.8864809,1.117929)
	F	0.06 (IQR 0.04-0.08) +<br>0.18 (IQR 0.07-0.34)	0.23 (95% 0.06-1.86 IQR 0.14-0.45)	dlnorm(-2.8690639,3.124268) +<br>dlnorm(-1.828793,0.653974)
Self-cure rate (annual)	M & F	0.20 (range 0.15-0.25)	0.19 (95% CrI 0.09-0.41 IQR 0.15-0.25)	dlnorm(-1.6417072,6.973734)
Untreated-TB mortality rate (annual)	M & F	0.32 (range 0.22-0.42)	0.30 (95% CrI 0.12-0.78 IQR 0.22-0.43)	dlnorm(-1.1908142,4.352139601)

M: male; F: female; CI: confidence interval; CrI: credible interval; IQR: inter-quartile range; Untreated disease duration: inverse of treatment initiation rate
* Modelled as proportion but shown as number per 100,000 population

WEB APPENDIX 2: DATA

Data for prevalence and case notification rates are summarised at the end of this section in

Web Table 6.

PREVALENCE

Prevalence estimates for smear-positive TB were taken from the most recent national prevalence survey conducted in each country. Weighted estimates were used to account for stratification by area, differential population growth prior to the survey and different cluster sizes.

In Viet Nam, a 2006-07 national prevalence survey reported weighted prevalence estimates for smear-positive TB of 351 (95% CI 262-440) per 100,000 men and 69 (95% CI 39-99) per 100,000 women (20). Treating uncertainty estimates as confidence intervals for a population mean, these data are approximated in the model by 59 cases per 16,809 male population and 20 cases per 28,986 female population, with a corresponding M:F ratio of 5.09 (95% CI 3.61-7.41).

In Malawi, a 2013-14 national prevalence survey reported weighted prevalence estimates for smear-positive TB of 303 (95% CI 176-431) per 100,000 men and 149 (95% CI 85-213) per 100,000 women (21). Treating uncertainty estimates as confidence intervals for a population mean, these data are approximated in the model by 21 cases per 6,931 male population and 21 cases per 14,094 female population, with a corresponding M:F ratio of 2.03 (95% CI 1.33-1.59).

The normal approximation to the binomial distribution was used to estimate the number of prevalent cases based on calculated prevalence and prevalence survey participants.

CASE NOTIFICATION RATE

Case notification counts for new cases of smear-positive TB in individuals ≥ 15 years of age were taken from data routinely reported by National TB Programmes to WHO (23). These data were matched to the nearest five-year national population estimate for individuals ≥ 15 years of age from the United Nations World Population Prospect (2, 3) to calculate annual case notification rates by gender.

In Viet Nam, the average number of annual case notifications reported over 2006-07 (the years of the prevalence survey) was 40,668 for men and 14,672 for women. The nearest population estimates from 2005 report a male population of 29,742,533 and a female population of 31,595,138. This gives a case notification rate (with confidence intervals ± 10% of the point estimate) of 137 (95% CI 123-151) per 100,000 men and 47 (95% CI 42-52) per 100,000 women. Treating uncertainty estimates as confidence intervals for a population mean, these data are approximated in the model by 350 cases in a male population of 255,474 and 300 cases in a female population of 638,298, with a corresponding M:F ratio of 2.92 (95% CI 2.61-3.26).

In Malawi, the average number of annual case notifications over 2011-12 (the last years in which case notifications for smear-positive TB were reported by gender) was 3,992 for men and 2,849 for women. The nearest population estimates from 2010 report a male population of 3,932,713 and a female population of 4,015,137. This gives a case notification rate (with confidence intervals ± 10% of the point estimate) of 102 (95% CI 91-112) per 100,000 men and 71 (95% CI 64-78) per 100,000 women. Treating uncertainty estimates as confidence intervals for a population mean, these data are approximated in the model by 400 cases in a male population of 392,157 and 400 cases in a female population of 563,380, with a corresponding M:F ratio of 2.03 (95% CI 1.30-1.59).

The normal approximation to the binomial distribution was used to estimate the number of notified cases based on calculated case notification rate and population.

Web Table 6: Data for prevalence and case notification rates for Viet Nam and Malawi

Viet Nam			
Parameter	Gender	Data<br>estimate (95% CI)	Data used in model<br>numerator / denominator
Prevalence<br>(per 100,000*)	M	351 (262-440)	59 / 16,809
	F	69 (39-99)	20 / 28,986
	M:F	5.09 (3.61-7.41)	-
Case notification rate<br>(per 100,000*)	M	137 (123-151)	350 / 255,474
	F	47 (42-52)	300 / 638,298
	M:F	2.03 (1.33-1.59)	-
Malawi			
Parameter	Gender	Data<br>estimate (95% CI)	Data used in model<br>numerator / denominator
Prevalence<br>(per 100,000*)	M	303 (176-431)	21 / 6,931
	F	149 (85-213)	21 / 14,094
	M:F	2.92 (2.61-3.26)	-
Case notification rate<br>(per 100,000*)	M	102 (91-112)	400 / 392,157
	F	71 (64-78)	400 / 563,380
	M:F	2.03 (1.30-1.59)	-

M: male; F: female; M:F: male-to-female ratio
* Modelled as proportion but shown as number per 100,000 population

WEB APPENDIX 3: DIRECT ACYCLIC GRAPHS

Model structure and corresponding equations are shown below in Web Web Figure ***1*** and Web Web Figure ***2***.

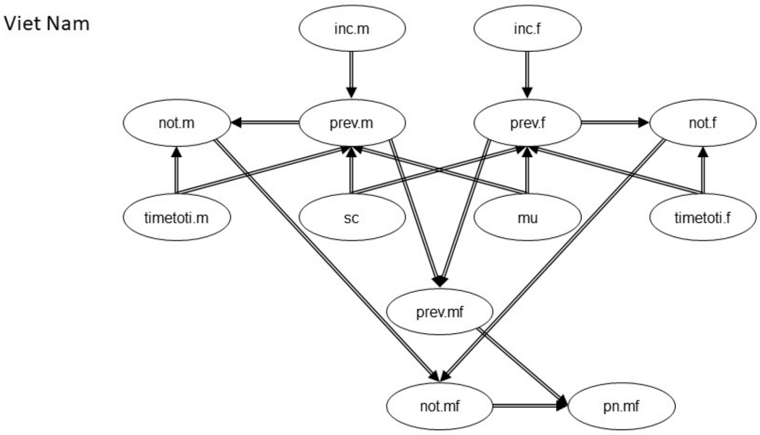

inc.m ~ dlnorm(-6.0126762,65.1874099)
inc.f ~ dlnorm(-7.4508502,6.5428704)
timetoti.m ~ dlnorm(-2.4474648,1.6973627)
timetoti.f ~ dlnorm(-2.2355818,2.4323685)
sc ~ dlnorm(-1.6417072,6.973734)
mu ~ dlnorm(-1.2261229,4.065313436)

prev.m = inc.m/((1/timetoti.m)+sc+mu)
prev.f = inc.f/((1/timetoti.f)+sc+mu)
not.m = (1/timetoti.m)*prev.m
not.f = (1/timetoti.f)*prev.f

prev.mf = prev.m/prev.f
not.mf = not.m/not.f
pn.mf = prev.mf/not.mf

Web Figure 1: Direct acyclic graph with corresponding equations for Viet Nam

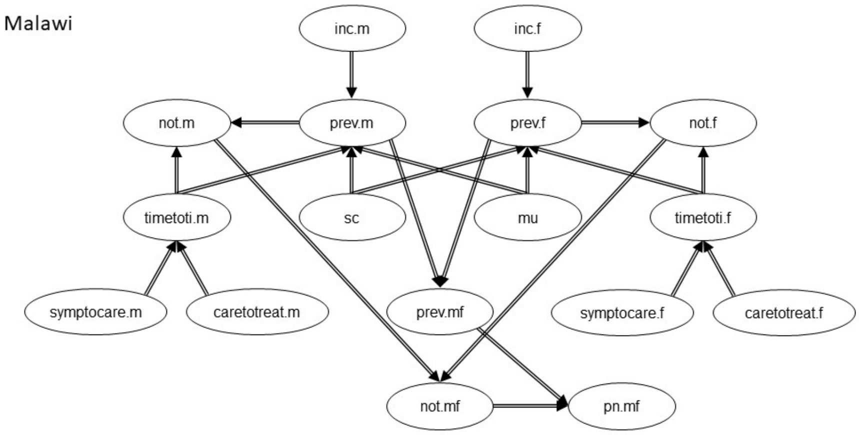

inc.m ~ dlnorm(-5.6429348,22.8071247)
inc.f ~ dlnorm(-6.4969191,4.0579866)
symptocare.m ~ dlnorm(-2.914242,3.788399)
caretotreat.m ~ dlnorm(-1.8864809,1.117929)
timetoti.m = symptocare.m + caretotreat.m
symptocare.f ~ dlnorm(-2.8690639,3.124268)
caretotreat.f ~ dlnorm(-1.828793,0.653974)
timetoti.f = symptocare.f + caretotreat.f
sc ~ dlnorm(-1.6417072,6.973734)
mu ~ dlnorm(-1.1908142,4.352139601)

prev.m = inc.m/((1/timetoti.m)+sc+mu)
prev.f = inc.f/((1/timetoti.f)+sc+mu)
not.m = (1/timetoti.m)*prev.m
not.f = (1/timetoti.f)*prev.f

prev.mf = prev.m/prev.f
not.mf = not.m/not.f
pn.mf = prev.mf/not.mf

Web Figure 2: Direct acyclic graph with corresponding equations for Malawi

WEB APPENDIX 4: CODE

The model is run using R (24), which calls WinBUGS (25) and associated data files. All files used in the main analysis and in sensitivity analyses are available at http://bit.ly/2HAKyon. A sample of the code used in the main analysis is included below for reference.

R CODE

```
## INSTALL PACKAGES ##

library(R2WinBUGS)
library(coda)

## PREPARE DATA AND PARAMETER LISTS ##

# Set working directory
setwd("C:/WinBUGSModel")

# Import data files
Data_VPr <- read.csv("C:/WinBUGSModel/Data_VPr.csv")
Data_VPo <- read.csv("C:/WinBUGSModel/Data_VPo.csv")
Data_MPr <- read.csv("C:/WinBUGSModel/Data_MPr.csv")
Data_MPo <- read.csv("C:/WinBUGSModel/Data_MPo.csv")

# List parameters
Params <-
c("inc.m","inc.f","timetoti.m","timetoti.f","sc","mu","prev.m","prev.f","not.m","not.f","prev.mf","not.
mf","pn.mf")

## RUN MODEL FOR VIET NAM ##

 # Priors (no confrontation)
 Model_V1PrW2A <- bugs(data=Data_VPr, inits=NULL, Params,
model.file="C:/WinBUGSModel/Model_VPr.txt", n.chains=3, n.iter=21000, n.burnin=1000,
n.thin=1, digits=5, bugs.directory="c:/WinBUGS14", DIC=FALSE)
 print(Model_V1PrW2A$summary,digits=5)
 gelman.diag(Model_V1PrW2A, confidence = 0.95, transform=FALSE, autoburnin=TRUE,
multivariate=TRUE)
 gelman.plot(Model_V1PrW2A)

 # Posteriors (after confrontation)
 Model_V1PoW2A <- bugs(data=Data_VPo, inits=NULL, Params,
model.file="C:/WinBUGSModel/Model_VPo.txt", n.chains=3, n.iter=21000, n.burnin=1000,
n.thin=1, digits=5, bugs.directory="c:/WinBUGS14", DIC=FALSE)
 print(Model_V1PoW2A$summary,digits=5)
 gelman.diag(Model_V1PoW2A, confidence = 0.95, transform=FALSE, autoburnin=TRUE,
multivariate=TRUE)
 gelman.plot(Model_V1PoW2A)
```

```
## RUN MODEL FOR MALAWI ##

 # Priors (no confrontation)
 Model_M1PrW2A <- bugs(data=Data_MPr, inits=NULL, Params,
model.file="C:/WinBUGSModel/Model_MPr.txt", n.chains=3, n.iter=21000, n.burnin=1000,
n.thin=1, digits=5, bugs.directory="c:/WinBUGS14", DIC=FALSE)
 print(Model_M1PrW2A$summary,digits=5)
 gelman.diag(Model_M1PrW2A, confidence = 0.95, transform=FALSE, autoburnin=TRUE,
multivariate=TRUE)
 gelman.plot(Model_M1PrW2A)

 # Posteriors (after confrontation)
 Model_M1PoW2A <- bugs(data=Data_MPo, inits=NULL, Params,
model.file="C:/WinBUGSModel/Model_MPo.txt", n.chains=3, n.iter=21000, n.burnin=1000,
n.thin=1, digits=5, bugs.directory="c:/WinBUGS14", DIC=FALSE)
 print(Model_M1PoW2A$summary,digits=5)
 gelman.diag(Model_M1PoW2A, confidence = 0.95, transform=FALSE, autoburnin=TRUE,
multivariate=TRUE)
 gelman.plot(Model_M1PoW2A)

sink()
```

WINBUGS CODE (MODEL_VPR.TXT)

```
model{

# Incidence

inc.m ~ dlnorm(-6.0126762,65.1874099)
inc.f ~ dlnorm(-7.4508502,6.5428704)

# Time to treatment initiation

timetoti.m ~ dlnorm(-2.4474648,1.6973627)
timetoti.f ~ dlnorm(-2.2355818,2.4323685)

# Self-cure

sc ~ dlnorm(-1.6417072,6.973734)

# Mortality

mu ~ dlnorm(-1.2261229,4.065313436)

# Calculate prevalence, case notification rate and M:F ratios

prev.m <- inc.m/((1/timetoti.m)+sc+mu)
prev.f <- inc.f/((1/timetoti.f)+sc+mu)

not.m <- (1/timetoti.m)*prev.m
```

```
not.f <- (1/timetoti.f)*prev.f

prev.mf <- prev.m/prev.f
not.mf <- not.m/not.f
pn.mf <- prev.mf/not.mf

# Calculate model priors

prevcasemodelprior.m ~ dbin(prev.m,prevpop.m)
notcasemodelprior.m ~ dbin(not.m,notpop.m)
prevcasemodelprior.f ~ dbin(prev.f,prevpop.f)
notcasemodelprior.f ~ dbin(not.f,notpop.f)

}
```

WINBUGS CODE (MODEL_VPO.TXT)

```
model{

# Incidence

inc.m ~ dlnorm(-6.0126762,65.1874099)
inc.f ~ dlnorm(-7.4508502,6.5428704)

# Time to treatment initiation

timetoti.m ~ dlnorm(-2.4474648,1.6973627)
timetoti.f ~ dlnorm(-2.2355818,2.4323685)

# Self-cure

sc ~ dlnorm(-1.6417072,6.973734)

# Mortality

mu ~ dlnorm(-1.2261229,4.065313436)

# Calculate prevalence, case notification rate and M:F ratios

prev.m <- inc.m/((1/timetoti.m)+sc+mu)
prev.f <- inc.f/((1/timetoti.f)+sc+mu)

not.m <- (1/timetoti.m)*prev.m
not.f <- (1/timetoti.f)*prev.f

prev.mf <- prev.m/prev.f
not.mf <- not.m/not.f
pn.mf <- prev.mf/not.mf

# Confront model with data on prevalence and case notification rate

prevcase.m ~ dbin(prev.m,prevpop.m)
prevcasemodelpost.m ~ dbin(prev.m,prevpop.m)
notcase.m ~ dbin(not.m,notpop.m)
notcasemodelpost.m ~ dbin(not.m,notpop.m)
```

```
prevcase.f ~ dbin(prev.f,prevpop.f)
prevcasemodelpost.f ~ dbin(prev.f,prevpop.f)
notcase.f ~ dbin(not.f,notpop.f)
notcasemodelpost.f ~ dbin(not.f,notpop.f)

}
```

WINBUGS CODE (MODEL_MPR.TXT)

```
model{

# Incidence

inc.m ~ dlnorm(-5.6429348,22.8071247)
inc.f ~ dlnorm(-6.4969191,4.0579866)

# Time to treatment initiation

symptocare.m ~ dlnorm(-2.914242,3.788399)
caretotreat.m ~ dlnorm(-1.8864809,1.117929)
timetoti.m <- symptocare.m + caretotreat.m
symptocare.f ~ dlnorm(-2.8690639,3.124268)
caretotreat.f ~ dlnorm(-1.828793,0.653974)
timetoti.f <- symptocare.f + caretotreat.f

# Self-cure

sc ~ dlnorm(-1.6417072,6.973734)

# Mortality

mu ~ dlnorm(-1.1908142,4.352139601)

# Calculate prevalence, case notification rate and M:F ratios

prev.m <- inc.m/((1/timetoti.m)+sc+mu)
prev.f <- inc.f/((1/timetoti.f)+sc+mu)

not.m <- (1/timetoti.m)*prev.m
not.f <- (1/timetoti.f)*prev.f

prev.mf <- prev.m/prev.f
not.mf <- not.m/not.f
pn.mf <- prev.mf/not.mf

# Calculate model priors

prevcasemodelprior.m ~ dbin(prev.m,prevpop.m)
notcasemodelprior.m ~ dbin(not.m,notpop.m)
prevcasemodelprior.f ~ dbin(prev.f,prevpop.f)
notcasemodelprior.f ~ dbin(not.f,notpop.f)

}
```

WINBUGS CODE (MODEL_MPO.TXT)

```
model{

# Incidence

inc.m ~ dlnorm(-5.6429348,22.8071247)
inc.f ~ dlnorm(-6.4969191,4.0579866)

# Time to treatment initiation

symptocare.m ~ dlnorm(-2.914242,3.788399)
caretotreat.m ~ dlnorm(-1.8864809,1.117929)
timetoti.m <- symptocare.m + caretotreat.m
symptocare.f ~ dlnorm(-2.8690639,3.124268)
caretotreat.f ~ dlnorm(-1.828793,0.653974)
timetoti.f <- symptocare.f + caretotreat.f

# Self-cure

sc ~ dlnorm(-1.6417072,6.973734)

# Mortality

mu ~ dlnorm(-1.1908142,4.352139601)

# Calculate prevalence, case notification rate and M:F ratios

prev.m <- inc.m/((1/timetoti.m)+sc+mu)
prev.f <- inc.f/((1/timetoti.f)+sc+mu)

not.m <- (1/timetoti.m)*prev.m
not.f <- (1/timetoti.f)*prev.f

prev.mf <- prev.m/prev.f
not.mf <- not.m/not.f
pn.mf <- prev.mf/not.mf

# Confront model with data on prevalence and case notification rate

prevcase.m ~ dbin(prev.m,prevpop.m)
prevcasemodelpost.m ~ dbin(prev.m,prevpop.m)
notcase.m ~ dbin(not.m,notpop.m)
notcasemodelpost.m ~ dbin(not.m,notpop.m)
prevcase.f ~ dbin(prev.f,prevpop.f)
prevcasemodelpost.f ~ dbin(prev.f,prevpop.f)
notcase.f ~ dbin(not.f,notpop.f)
notcasemodelpost.f ~ dbin(not.f,notpop.f)

}
```

DATA FILE (DATA_VPR.CSV)

```
prevpop.m,notpop.m,prevpop.f,notpop.f
16809,255474,28986,638298
```

DATA FILE (DATA_VPO.CSV)

A Bayesian approach to understanding gender differences in tuberculosis disease burden
Katherine C. Horton, Tom Sumner, Rein M. G. J. Houben, Elizabeth L. Corbett, Richard G. White

prevcase.m,prevpop.m,notcase.m,notpop.m,prevcase.f,prevpop.f,notcase.f,notpop.f
59,16809,350,255474,20,28986,300,638298

DATA FILE (DATA_MPR.CSV)

prevpop.m,notpop.m,prevpop.f,notpop.f
6931,392157,14094,563380

DATA FILE (DATA_MPO.CSV)

prevcase.m,prevpop.m,notcase.m,notpop.m,prevcase.f,prevpop.f,notcase.f,notpop.f
21,6931,400,392157,21,14094,400,563380

WEB APPENDIX 5: SENSITIVITY ANALYSES – MODEL STRUCTURE

We examined additional scenarios reflecting all combinations of fixing individual parameters by gender and allowing individual parameters to differ by gender, as shown in Web Table 7.

Web Table 7: Scenarios

Scenario	Incidence rate	Treatment initiation rate	Self-cure rate	Untreated-TB mortality rate
0	M=F	M=F	M=F	M=F
1A	M≠F	M=F	M=F	M=F
1B	M=F	M≠F	M=F	M=F
1C	M=F	M=F	M≠F	M=F
1D	M=F	M=F	M=F	M≠F
**MAIN**	**M≠F**	**M≠F**	**M=F**	**M=F**
2B	M≠F	M=F	M≠F	M=F
2C	M≠F	M=F	M=F	M≠F
2D	M=F	M≠F	M≠F	M=F
2E	M=F	M≠F	M=F	M≠F
2F	M=F	M=F	M≠F	M≠F
3A	M≠F	M≠F	M≠F	M=F
3B	M≠F	M≠F	M=F	M≠F
3C	M≠F	M=F	M≠F	M≠F
3D	M=F	M≠F	M≠F	M≠F
4	M≠F	M≠F	M≠F	M≠F

Only scenarios that allowed both disease incidence and treatment initiation rate to differ by gender were consistent with empirical data on gender-specific prevalence and case notification rates and M:F ratios in prevalence-to-notification ratios in both countries (Scenarios 3A, 3B, 4). Posterior estimates for incidence, treatment initiation, self-cure and untreated-TB mortality rates (and therefore prevalence and case notification rate) did not differ substantially between the main model and those that also allowed self-cure and/or untreated-TB mortality to differ by gender (Scenarios 3A, 3B, 4) in either country.

In Malawi, scenarios that allowed treatment initiation and untreated-TB mortality rates (Scenario 2E) or treatment initiation, self-cure and untreated-TB mortality rates to differ by gender (Scenario 3D) also were consistent with empirical data on gender-specific prevalence and case notification rates and M:F ratios in prevalence-to-notification ratios. In each of these models, posterior estimates for untreated-TB mortality rate among women were over twice those estimated among men. These differences were considered too extreme to be feasible in light of previous studies that have found no evidence of a gender difference in TB mortality (26).

Remaining scenarios did not generate posterior estimates consistent with observed gender-specific prevalence, case notification rates and/or with M:F ratios in prevalence or case notification rates.

SCENARIO 0

Scenario 0 examined the scenario in which no parameters were allowed to differ by gender. Modelled gender-specific prevalence and case notification rates were calculated as:

$$prev_g = \frac{inc}{ti + sc + mu}$$

$$not_g = prev_g * ti$$

The model could not generate posterior estimates consistent with empirical data on gender-specific prevalence and case notification rates, nor M:F ratios in prevalence-to-notification ratios, for either country (except female prevalence in Malawi). Results are shown below in Web Table 8.

Web Table 8: Prior and posterior estimates for Scenario 0

Viet Nam				
Parameter	Gender	Model priors median (95% CrI)	Empirical data estimate (95% CI)	Model posteriors median (95% CrI)
Incidence rate (annual per 100,000*)	M & F	160 (141-182)		159 (141-180)
Untreated disease duration (years)	M & F	0.09 (0.02-0.40)		2.19 (1.73-2.75)
Self-cure rate (annual)	M & F	0.19 (0.09-0.41)		0.21 (0.10-0.38)
Untreated-TB mortality rate (annual)	M & F	0.29 (0.11-0.77)		0.32 (0.15-0.52)
Prevalence (per 100,000*)	M		351 (262-440)	161 (129-198)
	F		69 (39-99)	161 (129-198)
Notification (per 100,000*)	M		137 (123-151)	73 (68-79)
	F		47 (42-52)	73 (68-79)
Prevalence-to-notification ratio	M:F		1.75 (1.21-2.58)	1.00 (1.00-1.00)
Malawi				
Parameter	Gender	Model priors median (95% CrI)	Empirical data estimate (95% CI)	Model posteriors median (95% CrI)
Incidence rate (annual per 100,000*)	M & F	288 (201-413)		239 (177-328)
Untreated disease duration (years)	M & F	0.22 (0.06-1.25)		2.39 (1.75-3.18)
Self-cure rate (annual)	M & F	0.19 (0.09-0.41)		0.23 (0.11-0.49)
Untreated-TB mortality rate (annual)	M & F	0.30 (0.12-0.78)		0.51 (0.21-1.03)
Prevalence (per 100,000*)	M		303 (176-431)	201 (149-265)
	F		149 (85-213)	201 (149-265)
Notification (per 100,000*)	M		102 (91-112)	84 (78-90)
	F		71 (64-78)	84 (78-90)
Prevalence-to-notification ratio	M:F		1.42 (0.91-2.20)	1.00 (1.00-1.00)

M: male; F: female; M:F: male-to-female ratio; CI: confidence interval; CrI: credible interval; Untreated disease duration: inverse of treatment initiation rate
*Modelled as proportion but shown as number per 100,000 population
All potential scale reduction factors, which equal one at convergence, were between 1.001 and 1.002.

SCENARIO 1A

In Scenario 1A, incidence rate was the only parameter allowed to differ by gender. Modelled gender-specific prevalence and case notification rates were calculated as:

$$prev_g = \frac{inc_g}{ti + sc + mu}$$

$$not_g = prev_g * ti$$

Results in Web Table 9 show that the model could not generate posterior estimates consistent with empirical data on M:F ratios in prevalence-to-notification ratios in either country, nor with male prevalence in Viet Nam.

Web Table 9: Prior and posterior estimates for Scenario 1A

Viet Nam				
Parameter	Gender	Model priors median (95% CrI)	Empirical data estimate (95% CI)	Model posteriors median (95% CrI)
Incidence rate (annual per 100,000*)	M	245 (192-312)		251 (211-304)
	F	58 (27-125)		81 (66-101)
Untreated disease duration (years)	M & F	0.09 (0.02-0.39)		1.93 (1.51-2.42)
Self-cure rate (annual)	M & F	0.19 (0.09-0.41)		0.17 (0.09-0.32)
Untreated-TB mortality rate (annual)	M & F	0.29 (0.11-0.77)		0.22 (0.10-0.42)
Prevalence (per 100,000*)	M		351 (262-440)	272 (216-338)
	F		69 (39-99)	88 (69-111)
Notification (per 100,000*)	M		137 (123-151)	141 (128-156)
	F		47 (42-52)	46 (41-51)
Prevalence-to-notification ratio	M:F		1.75 (1.21-2.58)	1.00 (1.00-1.00)
Malawi				
Parameter	Gender	Model priors median (95% CrI)	Empirical data estimate (95% CI)	Model posteriors median (95% CrI)
Incidence rate (annual per 100,000*)	M	354 (234-533)		277 (208-386)
	F	152 (57-401)		186 (139-264)
Untreated disease duration (years)	M & F	0.22 (0.06-1.22)		2.44 (1.79-3.23)
Self-cure rate (annual)	M & F	0.19 (0.09-0.41)		0.22 (0.10-0.46)
Untreated-TB mortality rate (annual)	M & F	0.30 (0.12-0.78)		0.44 (0.19-0.90)
Prevalence (per 100,000*)	M		303 (176-431)	254 (186-336)
	F		149 (85-213)	171 (126-226)
Notification (per 100,000*)	M		102 (91-112)	104 (95-115)
	F		71 (64-78)	70 (64-77)
Prevalence-to-notification ratio	M:F		1.42 (0.91-2.20)	1.00 (1.00-1.00)

M: male; F: female; M:F: male-to-female ratio; CI: confidence interval; CrI: credible interval; Untreated disease duration: inverse of treatment initiation rate
*Modelled as proportion but shown as number per 100,000 population
All potential scale reduction factors, which equal one at convergence, were between 1.000 and 1.003.

SCENARIO 1B

In Scenario 1B, treatment initiation rate was the only parameter allowed to differ by gender. Modelled gender-specific prevalence and case notification rates were calculated as:

$$prev_g = \frac{inc}{ti_g + sc + mu}$$

$$not_g = prev_g * ti_g$$

Results in Web Table 10 show that the model could not generate posterior estimates consistent with empirical data on prevalence, nor M:F ratios in prevalence-to-notification ratios, in either country.

Web Table 10: Prior and posterior estimates for Scenario 1B

Viet Nam				
Parameter	Gender	Model priors median (95% CrI)	Empirical data estimate (95% CI)	Model posteriors median (95% CrI)
Incidence rate (annual per 100,000*)	M & F	160 (141-182)		190 (171-212)
Untreated disease duration (years)	M	0.09 (0.02-0.39)		0.85 (0.63-1.13)
	F	0.11 (0.03-0.38)		3.62 (2.79-4.62)
Self-cure rate (annual)	M & F	0.19 (0.09-0.41)		0.24 (0.11-0.49)
Untreated-TB mortality rate (annual)	M & F	0.30 (0.11-0.78)		0.51 (0.24-0.80)
Prevalence (per 100,000*)	M		351 (262-440)	98 (75-125)
	F		69 (39-99)	183 (146-227)
Notification (per 100,000*)	M		137 (123-151)	115 (104-127)
	F		47 (42-52)	51 (45-57)
Prevalence-to-notification ratio	M:F		1.75 (1.21-2.58)	0.24 (0.18-0.31)
Malawi				
Parameter	Gender	Model priors median (95% CrI)	Empirical data estimate (95% CI)	Model posteriors median (95% CrI)
Incidence rate (annual per 100,000*)	M & F	288 (201-414)		244 (185-331)
Untreated disease duration (years)	M	0.22 (0.07-1.03)		1.68 (1.18-2.32)
	F	0.23 (0.06-1.86)		2.86 (2.08-3.84)
Self-cure rate (annual)	M & F	0.19 (0.09-0.41)		0.24 (0.11-0.52)
Untreated-TB mortality rate (annual)	M & F	0.30 (0.12-0.77)		0.58 (0.24-1.12)
Prevalence (per 100,000*)	M		303 (176-431)	171 (123-229)
	F		149 (85-213)	207 (154-271)
Notification (per 100,000*)	M		102 (91-112)	102 (92-112)
	F		71 (64-78)	72 (65-79)
Prevalence-to-notification ratio	M:F		1.42 (0.91-2.20)	0.59 (0.46-0.73)

M: male; F: female; M:F: male-to-female ratio; CI: confidence interval; CrI: credible interval; Untreated disease duration: inverse of treatment initiation rate

*Modelled as proportion but shown as number per 100,000 population

All potential scale reduction factors, which equal one at convergence, were between 1.001 and 1.002.

SCENARIO 1C

In Scenario 1C, self-cure rate was the only parameter allowed to differ by gender. Modelled gender-specific prevalence and case notification rates were calculated as:

$$prev_g = \frac{inc}{ti + sc_g + mu}$$

$$not_g = prev_g * ti$$

Results in Web Table 11 show that the model could not generate posterior estimates consistent with empirical data on M:F ratios in prevalence-to-notification ratios in either country, nor female prevalence in Viet Nam.

Web Table 11: Prior and posterior estimates for Scenario 1C

Viet Nam				
Parameter	Gender	Model priors median (95% CrI)	Empirical data estimate (95% CI)	Model posteriors median (95% CrI)
Incidence rate (annual per 100,000*)	M & F	160 (141-182)		175 (159-194)
Untreated disease duration (years)	M & F	0.09 (0.02-0.39)		2.13 (1.7-2.65)
Self-cure rate (annual)	M	0.19 (0.09-0.41)		0.09 (0.05-0.14)
	F	0.19 (0.09-0.41)		1.14 (0.87-1.50)
Untreated-TB mortality rate (annual)	M & F	0.30 (0.11-0.78)		0.09 (0.05-0.16)
Prevalence (per 100,000*)	M		351 (262-440)	269 (216-332)
	F		69 (39-99)	103 (82-128)
Notification (per 100,000*)	M		137 (123-151)	126 (115-139)
	F		47 (42-52)	48 (43-54)
Prevalence-to-notification ratio	M:F		1.75 (1.21-2.58)	1.00 (1.00-1.00)
Malawi				
Parameter	Gender	Model priors median (95% CrI)	Empirical data estimate (95% CI)	Model posteriors median (95% CrI)
Incidence rate (annual per 100,000*)	M & F	289 (201-414)		232 (179-308)
Untreated disease duration (years)	M & F	0.22 (0.06-1.22)		2.55 (1.89-3.35)
Self-cure rate (annual)	M	0.19 (0.09-0.41)		0.15 (0.08-0.27)
	F	0.19 (0.09-0.41)		0.48 (0.31-0.72)
Untreated-TB mortality rate (annual)	M & F	0.30 (0.12-0.78)		0.36 (0.16-0.70)
Prevalence (per 100,000*)	M		303 (176-431)	254 (187-335)
	F		149 (85-213)	187 (139-243)
Notification (per 100,000*)	M		102 (91-112)	100 (90-110)
	F		71 (64-78)	73 (66-80)
Prevalence-to-notification ratio	M:F		1.42 (0.91-2.20)	1.00 (1.00-1.00)

M: male; F: female; M:F: male-to-female ratio; CI: confidence interval; CrI: credible interval; Untreated disease duration: inverse of treatment initiation rate
*Modelled as proportion but shown as number per 100,000 population
All potential scale reduction factors, which equal one at convergence, were between 1.000 and 1.002.

SCENARIO 1D

In Scenario 1D, untreated-TB mortality rate was the only parameter allowed to differ by gender. Modelled gender-specific prevalence and case notification rates were calculated as:

$$prev_g = \frac{inc}{ti + sc + mu_g}$$

$$not_g = prev_g * ti$$

Results in Web Table 12 show that the model could not generate posterior estimates consistent with empirical data on M:F ratios in prevalence-to-notification ratios in either country, nor male or female prevalence in Viet Nam.

Web Table 12: Prior and posterior estimates for Scenario 1D

Parameter	Gender	Model priors median (95% CrI)	Empirical data estimate (95% CI)	Model posteriors median (95% CrI)
Viet Nam				
Incidence rate (annual per 100,000*)	M & F	160 (141-182)		179 (162-198)
Untreated disease duration (years)	M & F	0.09 (0.02-0.39)		1.98 (1.56-2.47)
Self-cure rate (annual)	M & F	0.19 (0.09-0.41)		0.10 (0.06-0.17)
Untreated-TB mortality rate (annual)	M	0.29 (0.11-0.77)		0.09 (0.05-0.16)
	F	0.30 (0.11-0.78)		1.31 (0.98-1.74)
Prevalence (per 100,000*)	M		351 (262-440)	255 (204-317)
	F		69 (39-99)	93 (74-117)
Notification (per 100,000*)	M		137 (123-151)	129 (117-142)
	F		47 (42-52)	47 (42-53)
Prevalence-to-notification ratio	M:F		1.75 (1.21-2.58)	1.00 (1.00-1.00)
Malawi				
Parameter	Gender	Model priors median (95% CrI)	Empirical data estimate (95% CI)	Model posteriors median (95% CrI)
Incidence rate (annual per 100,000*)	M & F	289 (201-414)		234 (182-306)
Untreated disease duration (years)	M & F	0.22 (0.06-1.22)		2.5 (1.86-3.3)
Self-cure rate (annual)	M & F	0.19 (0.09-0.41)		0.23 (0.11-0.46)
Untreated-TB mortality rate (annual)	M	0.30 (0.12-0.78)		0.27 (0.12-0.52)
	F	0.30 (0.12-0.78)		0.67 (0.41-1.05)
Prevalence (per 100,000*)	M		303 (176-431)	256 (190-338)
	F		149 (85-213)	179 (134-235)
Notification (per 100,000*)	M		102 (91-112)	102 (93-112)
	F		71 (64-78)	72 (65-79)
Prevalence-to-notification ratio	M:F		1.42 (0.91-2.20)	1.00 (1.00-1.00)

M: male; F: female; M:F: male-to-female ratio; CI: confidence interval; CrI: credible interval; Untreated disease duration: inverse of treatment initiation rate
*Modelled as proportion but shown as number per 100,000 population
All potential scale reduction factors, which equal one at convergence, were between 1.000 and 1.002.

SCENARIO 2B

In Scenario 2B, incidence and self-cure rates were allowed to differ by gender. Modelled gender-specific prevalence and case notification rates were calculated as:

$$prev_g = \frac{inc_g}{ti + sc_g + mu}$$

$$not_g = prev_g * ti$$

Results in Web Table 13 show that the model could not generate posterior estimates consistent with empirical data on M:F ratios in prevalence-to-notification ratios in either country, nor male prevalence in Viet Nam.

Web Table 13: Prior and posterior estimates for Scenario 2B

Parameter	Gender	Model priors median (95% CrI)	Empirical data estimate (95% CI)	Model posteriors median (95% CrI)
Viet Nam				
Incidence rate (annual per 100,000*)	M	245 (192-312)		253 (212-307)
	F	58 (27-124)		82 (65-106)
Untreated disease duration (years)	M & F	0.09 (0.02-0.39)		1.92 (1.51-2.42)
Self-cure rate (annual)	M	0.19 (0.09-0.41)		0.18 (0.09-0.34)
	F	0.19 (0.09-0.41)		0.18 (0.09-0.36)
Untreated-TB mortality rate (annual)	M & F	0.29 (0.11-0.77)		0.22 (0.10-0.41)
Prevalence (per 100,000*)	M		351 (262-440)	271 (215-338)
	F		69 (39-99)	88 (68-112)
Notification (per 100,000*)	M		137 (123-151)	141 (128-155)
	F		47 (42-52)	46 (41-51)
Prevalence-to-notification ratio	M:F		1.75 (1.21-2.58)	1.00 (1.00-1.00)
Malawi				
Parameter	Gender	Model priors median (95% CrI)	Empirical data estimate (95% CI)	Model posteriors median (95% CrI)
Incidence rate (annual per 100,000*)	M	355 (235-536)		279 (207-387)
	F	150 (57-400)		180 (128-260)
Untreated disease duration (years)	M & F	0.22 (0.06-1.22)		2.43 (1.79-3.21)
Self-cure rate (annual)	M	0.19 (0.09-0.40)		0.23 (0.11-0.49)
	F	0.19 (0.09-0.41)		0.19 (0.09-0.39)
Untreated-TB mortality rate (annual)	M & F	0.30 (0.12-0.77)		0.44 (0.18-0.90)
Prevalence (per 100,000*)	M		303 (176-431)	253 (187-335)
	F		149 (85-213)	171 (126-225)
Notification (per 100,000*)	M		102 (91-112)	104 (95-114)
	F		71 (64-78)	70 (64-77)
Prevalence-to-notification ratio	M:F		1.42 (0.91-2.20)	1.00 (1.00-1.00)

M: male; F: female; M:F: male-to-female ratio; CI: confidence interval; CrI: credible interval; Untreated disease duration: inverse of treatment initiation rate
*Modelled as proportion but shown as number per 100,000 population
All potential scale reduction factors, which equal one at convergence, were between 1.000 and 1.002.

SCENARIO 2C

In Scenario 2C, incidence and untreated-TB mortality rates were allowed to differ by gender. Modelled gender-specific prevalence and case notification rates were calculated as:

$$prev_g = \frac{inc_g}{ti + sc + mu_g}$$

$$not_g = prev_g * ti$$

Results in Web Table 14 show that the model could not generate posterior estimates consistent with empirical data on M:F ratios in prevalence-to-notification ratios in either country, nor male prevalence in Viet Nam.

Web Table 14: Prior and posterior estimates for Scenario 2C

Parameter	Gender	Model priors median (95% CrI)	Empirical data estimate (95% CI)	Model posteriors median (95% CrI)
Viet Nam				
Incidence rate (annual per 100,000*)	M	245 (192-312)		255 (213-309)
	F	58 (27-124)		83 (65-115)
Untreated disease duration (years)	M & F	0.09 (0.02-0.39)		1.92 (1.51-2.40)
Self-cure rate (annual)	M & F	0.19 (0.09-0.41)		0.17 (0.09-0.32)
Untreated-TB mortality rate (annual)	M	0.29 (0.11-0.78)		0.24 (0.11-0.45)
	F	0.29 (0.11-0.77)		0.25 (0.10-0.57)
Prevalence (per 100,000*)	M		351 (262-440)	270 (214-336)
	F		69 (39-99)	88 (68-110)
Notification (per 100,000*)	M		137 (123-151)	141 (128-156)
	F		47 (42-52)	46 (41-51)
Prevalence-to-notification ratio	M:F		1.75 (1.21-2.58)	1.00 (1.00-1.00)
Malawi				
Parameter	Gender	Model priors median (95% CrI)	Empirical data estimate (95% CI)	Model posteriors median (95% CrI)
Incidence rate (annual per 100,000*)	M	355 (235-536)		285 (209-398)
	F	150 (57-400)		162 (117-242)
Untreated disease duration (years)	M & F	0.22 (0.06-1.22)		2.44 (1.80-3.22)
Self-cure rate (annual)	M & F	0.19 (0.09-0.40)		0.22 (0.10-0.46)
Untreated-TB mortality rate (annual)	M	0.30 (0.12-0.78)		0.47 (0.20-0.96)
	F	0.30 (0.12-0.77)		0.29 (0.12-0.69)
Prevalence (per 100,000*)	M		303 (176-431)	253 (187-334)
	F		149 (85-213)	171 (126-226)
Notification (per 100,000*)	M		102 (91-112)	104 (94-114)
	F		71 (64-78)	70 (64-77)
Prevalence-to-notification ratio	M:F		1.42 (0.91-2.20)	1.00 (1.00-1.00)

M: male; F: female; M:F: male-to-female ratio; CI: confidence interval; CrI: credible interval; Untreated disease duration: inverse of treatment initiation rate
*Modelled as proportion but shown as number per 100,000 population
All potential scale reduction factors, which equal one at convergence, were between 1.000 and 1.003.

SCENARIO 2D

In Scenario 2D, treatment initiation and self-cure rates were allowed to differ by gender. Modelled gender-specific prevalence and case notification rates were calculated as:

$$prev_g = \frac{inc}{ti_g + sc_g + mu}$$

$$not_g = prev_g * ti$$

Results in Web Table 15 show that the model could not generate posterior estimates consistent with empirical data on male prevalence or M:F ratios in prevalence-to-notification ratios in either country.

Web Table 15: Prior and posterior estimates for Scenario 2D

Viet Nam				
Parameter	Gender	Model priors median (95% CrI)	Empirical data estimate (95% CI)	Model posteriors median (95% CrI)
Incidence rate (annual per 100,000*)	M & F	160 (141-182)		175 (159-193)
Untreated disease duration (years)	M	0.09 (0.02-0.39)		1.97 (1.45-2.63)
	F	0.11 (0.03-0.38)		1.90 (1.32-2.66)
Self-cure rate (annual)	M	0.19 (0.09-0.41)		0.09 (0.05-0.15)
	F	0.19 (0.09-0.41)		1.26 (0.87-1.85)
Untreated-TB mortality rate (annual)	M & F	0.29 (0.11-0.78)		0.09 (0.05-0.17)
Prevalence (per 100,000*)	M		351 (262-440)	251 (188-328)
	F		69 (39-99)	93 (66-127)
Notification (per 100,000*)	M		137 (123-151)	128 (116-140)
	F		47 (42-52)	49 (44-55)
Prevalence-to-notification ratio	M:F		1.75 (1.21-2.58)	1.04 (0.66-1.66)
Malawi				
Parameter	Gender	Model priors median (95% CrI)	Empirical data estimate (95% CI)	Model posteriors median (95% CrI)
Incidence rate (annual per 100,000*)	M & F	288 (201-414)		235 (178-320)
Untreated disease duration (years)	M	0.22 (0.07-1.03)		1.93 (1.27-2.93)
	F	0.23 (0.06-1.86)		2.70 (1.93-3.68)
Self-cure rate (annual)	M	0.19 (0.09-0.41)		0.17 (0.08-0.32)
	F	0.19 (0.09-0.41)		0.32 (0.14-0.65)
Untreated-TB mortality rate (annual)	M & F	0.30 (0.12-0.78)		0.50 (0.20-1.01)
Prevalence (per 100,000*)	M		303 (176-431)	197 (132-294)
	F		149 (85-213)	195 (142-259)
Notification (per 100,000*)	M		102 (91-112)	102 (92-112)
	F		71 (64-78)	72 (65-79)
Prevalence-to-notification ratio	M:F		1.42 (0.91-2.20)	0.71 (0.49-1.14)

M: male; F: female; M:F: male-to-female ratio; CI: confidence interval; CrI: credible interval; Untreated disease duration: inverse of treatment initiation rate
*Modelled as proportion but shown as number per 100,000 population
All potential scale reduction factors, which equal one at convergence, were between 1.001 and 1.004.

SCENARIO 2E

In Scenario 2E, treatment initiation and untreated-TB mortality rates were allowed to differ by gender. Modelled gender-specific prevalence and case notification rates were calculated as:

$$prev_g = \frac{inc}{ti_g + sc + mu_g}$$

$$not_g = prev_g * ti_g$$

Results in Web Table 16 show that the model could not generate posterior estimates consistent with empirical data on male prevalence in Viet Nam. The model produced posterior estimates that were consistent with empirical data on gender-specific prevalence and case notification rates and M:F ratios in prevalence-to-notification ratios in Malawi. However, posterior estimates for untreated-TB mortality rate among women were over twice those estimated among men, which is inconsistent with available evidence (26).

Web Table 16: Prior and posterior estimates for Scenario 2E

Parameter	Gender	Model priors median (95% CrI)	Empirical data estimate (95% CI)	Model posteriors median (95% CrI)
**Viet Nam**				
Incidence rate (annual per 100,000*)	M & F	160 (141-182)		178 (162-197)
Untreated disease duration (years)	M	0.09 (0.02-0.39)		1.99 (1.47-2.65)
	F	0.11 (0.03-0.38)		1.49 (0.99-2.17)
Self-cure rate (annual)	M & F	0.19 (0.09-0.41)		0.10 (0.06-0.16)
Untreated-TB mortality rate (annual)	M	0.29 (0.11-0.77)		0.09 (0.05-0.16)
	F	0.29 (0.11-0.78)		1.70 (1.13-2.60)
Prevalence (per 100,000*)	M		351 (262-440)	257 (193-334)
	F		69 (39-99)	72 (49-103)
Notification (per 100,000*)	M		137 (123-151)	129 (117-142)
	F		47 (42-52)	48 (43-54)
Prevalence-to-notification ratio	M:F		1.75 (1.21-2.58)	1.34 (0.82-2.21)
**Malawi**				
Parameter	Gender	Model priors median (95% CrI)	Empirical data estimate (95% CI)	Model posteriors median (95% CrI)
Incidence rate (annual per 100,000*)	M & F	288 (201-414)		229 (178-301)
Untreated disease duration (years)	M	0.22 (0.07-1.03)		2.35 (1.52-3.5)
	F	0.23 (0.06-1.86)		2.36 (1.59-3.39)
Self-cure rate (annual)	M & F	0.19 (0.09-0.41)		0.23 (0.11-0.47)
Untreated-TB mortality rate (annual)	M	0.30 (0.12-0.78)		0.27 (0.12-0.57)
	F	0.30 (0.12-0.78)		0.68 (0.34-1.24)
Prevalence (per 100,000*)	M		303 (176-431)	241 (158-355)
	F		149 (85-213)	169 (115-239)
Notification (per 100,000*)	M		102 (91-112)	103 (93-113)
	F		71 (64-78)	72 (65-79)
Prevalence-to-notification ratio	M:F		1.42 (0.91-2.20)	0.99 (0.58-1.71)

M: male; F: female; M:F: male-to-female ratio; CI: confidence interval; CrI: credible interval; Untreated disease duration: inverse of treatment initiation rate
*Modelled as proportion but shown as number per 100,000 population
All potential scale reduction factors, which equal one at convergence, were between 1.001 and 1.004.

SCENARIO 2F

In Scenario 2F, self-cure and untreated-TB mortality rates were allowed to differ by gender. Modelled gender-specific prevalence and case notification rates were calculated as:

$$prev_g = \frac{inc}{ti + sc_g + mu_g}$$

$$not_g = prev_g * ti$$

Results in Web Table 17 show that the model could not generate posterior estimates consistent with empirical data on M:F ratios in prevalence-to-notification ratios in either country, nor male or female prevalence in Viet Nam.

Web Table 17: Prior and posterior estimates for Scenario 2F

Viet Nam				
Parameter	Gender	Model priors median (95% CrI)	Empirical data estimate (95% CI)	Model posteriors median (95% CrI)
Incidence rate (annual per 100,000*)	M & F	160 (141-182)		178 (162-197)
Untreated disease duration (years)	M & F	0.09 (0.02-0.39)		1.98 (1.57-2.48)
Self-cure rate (annual)	M	0.19 (0.09-0.41)		0.10 (0.05-0.16)
	F	0.19 (0.09-0.41)		0.24 (0.11-0.57)
Untreated-TB mortality rate (annual)	M	0.29 (0.11-0.77)		0.09 (0.05-0.16)
	F	0.29 (0.11-0.78)		1.14 (0.69-1.62)
Prevalence (per 100,000*)	M		351 (262-440)	257 (205-318)
	F		69 (39-99)	94 (74-118)
Notification (per 100,000*)	M		137 (123-151)	129 (118-142)
	F		47 (42-52)	47 (42-53)
Prevalence-to-notification ratio	M:F		1.75 (1.21-2.58)	1.00 (1.00-1.00)
Malawi				
Parameter	Gender	Model priors median (95% CrI)	Empirical data estimate (95% CI)	Model posteriors median (95% CrI)
Incidence rate (annual per 100,000*)	M & F	288 (201-413)		231 (180-300)
Untreated disease duration (years)	M & F	0.22 (0.06-1.22)		2.52 (1.86-3.31)
Self-cure rate (annual)	M	0.19 (0.09-0.40)		0.19 (0.09-0.37)
	F	0.19 (0.09-0.41)		0.24 (0.11-0.54)
Untreated-TB mortality rate (annual)	M	0.29 (0.11-0.77)		0.29 (0.13-0.55)
	F	0.29 (0.11-0.78)		0.62 (0.29-1.07)
Prevalence (per 100,000*)	M		303 (176-431)	258 (190-339)
	F		149 (85-213)	180 (133-235)
Notification (per 100,000*)	M		102 (91-112)	103 (93-113)
	F		71 (64-78)	71 (65-78)
Prevalence-to-notification ratio	M:F		1.42 (0.91-2.20)	1.00 (1.00-1.00)

M: male; F: female; M:F: male-to-female ratio; CI: confidence interval; CrI: credible interval; Untreated disease duration: inverse of treatment initiation rate
*Modelled as proportion but shown as number per 100,000 population
All potential scale reduction factors, which equal one at convergence, were between 1.000 and 1.002.

SCENARIO 3A

In Scenario 3A, incidence, treatment initiation and self-cure rates were allowed to differ by gender. Modelled gender-specific prevalence and case notification rates were calculated as:

$$prev_g = \frac{inc_g}{ti_g + sc_g + mu}$$

$$not_g = prev_g * ti_g$$

Results in Web Table 18 show that the model produced posterior estimates that were consistent with empirical data on gender-specific prevalence and case notification rates and M:F ratios in prevalence-to-notification ratios in both countries. Posterior estimates for transition rates, and therefore prevalence and case notification rate, do not differ substantially from the main model.

Web Table 18: Prior and posterior estimates for Scenario 3A

Viet Nam				
Parameter	Gender	Model priors median (95% CrI)	Empirical data estimate (95% CI)	Model posteriors median (95% CrI)
Incidence rate (annual per 100,000*)	M	245 (192-313)		259 (216-314)
	F	58 (27-125)		68 (56-86)
Untreated disease duration (years)	M	0.09 (0.02-0.39)		2.20 (1.65-2.89)
	F	0.11 (0.03-0.38)		1.01 (0.60-1.59)
Self-cure rate (annual)	M	0.19 (0.09-0.40)		0.17 (0.09-0.32)
	F	0.19 (0.09-0.41)		0.19 (0.09-0.39)
Untreated-TB mortality rate (annual)	M & F	0.30 (0.11-0.78)		0.21 (0.10-0.40)
Prevalence (per 100,000*)	M		351 (262-440)	305 (234-390)
	F		69 (39-99)	48 (29-75)
Notification (per 100,000*)	M		137 (123-151)	138 (125-153)
	F		47 (42-52)	48 (43-53)
Prevalence-to-notification ratio	M:F		1.75 (1.21-2.58)	2.19 (1.28-3.90)
Malawi				
Parameter	Gender	Model priors median (95% CrI)	Empirical data estimate (95% CI)	Model posteriors median (95% CrI)
Incidence rate (annual per 100,000*)	M	355 (235-536)		295 (215-415)
	F	152 (57-396)		155 (112-232)
Untreated disease duration (years)	M	0.22 (0.07-1.03)		2.78 (1.82-4.08)
	F	0.23 (0.06-1.89)		1.88 (1.17-2.85)
Self-cure rate (annual)	M	0.19 (0.09-0.41)		0.22 (0.10-0.47)
	F	0.19 (0.09-0.41)		0.19 (0.09-0.39)
Untreated-TB mortality rate (annual)	M & F	0.30 (0.12-0.78)		0.42 (0.18-0.86)
Prevalence (per 100,000*)	M		303 (176-431)	287 (190-415)
	F		149 (85-213)	134 (84-200)
Notification (per 100,000*)	M		102 (91-112)	103 (93-114)
	F		71 (64-78)	71 (65-78)
Prevalence-to-notification ratio	M:F		1.42 (0.91-2.20)	1.49 (0.82-2.69)

M: male; F: female; M:F: male-to-female ratio; CI: confidence interval; CrI: credible interval; Untreated disease duration: inverse of treatment initiation rate
*Modelled as proportion but shown as number per 100,000 population

All potential scale reduction factors, which equal one at convergence, were between 1.001 and 1.003.

SCENARIO 3B

In Scenario 3B, incidence, treatment initiation and untreated-TB mortality rates were allowed to differ by gender. Modelled gender-specific prevalence and case notification rates were calculated as:

$$prev_g = \frac{inc_g}{ti_g + sc + mu_g}$$

$$not_g = prev_g * ti_g$$

Results in Web Table 19 show that the model produced posterior estimates that were consistent with empirical data on gender-specific prevalence and case notification rates and M:F ratios in prevalence-to-notification ratios in both countries. Posterior estimates for transition rates, and therefore prevalence and case notification rate, do not differ substantially from the main model.

Web Table 19: Prior and posterior estimates for Scenario 3B

Viet Nam				
Parameter	Gender	Model priors median (95% CrI)	Empirical data estimate (95% CI)	Model posteriors median (95% CrI)
Incidence rate (annual per 100,000*)	M	245 (192-313)		259 (215-315)
	F	58 (27-125)		69 (57-93)
Untreated disease duration (years)	M	0.09 (0.02-0.39)		2.19 (1.65-2.88)
	F	0.11 (0.03-0.38)		1.00 (0.60-1.58)
Self-cure rate (annual)	M & F	0.19 (0.09-0.40)		0.17 (0.09-0.31)
Untreated-TB mortality rate (annual)	M	0.29 (0.11-0.77)		0.22 (0.10-0.42)
	F	0.30 (0.11-0.78)		0.27 (0.11-0.67)
Prevalence (per 100,000*)	M		351 (262-440)	304 (233-389)
	F		69 (39-99)	48 (29-74)
Notification (per 100,000*)	M		137 (123-151)	138 (125-153)
	F		47 (42-52)	48 (43-53)
Prevalence-to-notification ratio	M:F		1.75 (1.21-2.58)	2.19 (1.28-3.92)
Malawi				
Parameter	Gender	Model priors median (95% CrI)	Empirical data estimate (95% CI)	Model posteriors median (95% CrI)
Incidence rate (annual per 100,000*)	M	355 (235-536)		297 (216-420)
	F	151 (57-396)		145 (106-218)
Untreated disease duration (years)	M	0.22 (0.07-1.03)		2.76 (1.80-4.06)
	F	0.23 (0.06-1.89)		1.89 (1.18-2.84)
Self-cure rate (annual)	M & F	0.19 (0.09-0.41)		0.22 (0.10-0.46)
Untreated-TB mortality rate (annual)	M	0.30 (0.12-0.78)		0.44 (0.18-0.92)
	F	0.30 (0.12-0.78)		0.31 (0.12-0.73)
Prevalence (per 100,000*)	M		303 (176-431)	284 (188-414)
	F		149 (85-213)	135 (85-200)
Notification (per 100,000*)	M		102 (91-112)	103 (94-114)

	F		71 (64-78)	71 (65-78)
Prevalence-to-notification ratio	M:F		1.42 (0.91-2.20)	1.46 (0.81-2.68)

M: male; F: female; M:F: male-to-female ratio; CI: confidence interval; CrI: credible interval; Untreated disease duration: inverse of treatment initiation rate
*Modelled as proportion but shown as number per 100,000 population
All potential scale reduction factors, which equal one at convergence, were between 1.001 and 1.004.

SCENARIO 3C

In Scenario 3C, incidence, self-cure and untreated-TB mortality rates were allowed to differ by gender. Modelled gender-specific prevalence and case notification rates were calculated as:

$$prev_g = \frac{inc_g}{ti + sc_g + mu_g}$$

$$not_g = prev_g * ti$$

Results in Web Table 20 show that the model could not generate posterior estimates consistent with empirical data on M:F ratios in prevalence-to-notification ratios in either country, nor male prevalence in Viet Nam.

Web Table 20: Prior and posterior estimates for Scenario 3C

Viet Nam				
Parameter	Gender	Model priors median (95% CrI)	Empirical data estimate (95% CI)	Model posteriors median (95% CrI)
Incidence rate (annual per 100,000*)	M	245 (192-313)		256 (214-311)
	F	58 (27-125)		85 (65-118)
Untreated disease duration (years)	M & F	0.09 (0.02-0.39)		1.91 (1.50-2.40)
Self-cure rate (annual)	M	0.19 (0.09-0.41)		0.18 (0.09-0.33)
	F	0.19 (0.09-0.40)		0.18 (0.09-0.37)
Untreated-TB mortality rate (annual)	M	0.29 (0.11-0.77)		0.23 (0.11-0.45)
	F	0.30 (0.11-0.78)		0.25 (0.10-0.58)
Prevalence (per 100,000*)	M		351 (262-440)	269 (214-334)
	F		69 (39-99)	87 (68-110)
Notification (per 100,000*)	M		137 (123-151)	141 (128-155)
	F		47 (42-52)	46 (41-51)
Prevalence-to-notification ratio	M:F		1.75 (1.21-2.58)	1.00 (1.00-1.00)
Malawi				
Parameter	Gender	Model priors median (95% CrI)	Empirical data estimate (95% CI)	Model posteriors median (95% CrI)
Incidence rate (annual per 100,000*)	M	354 (234-536)		286 (210-402)
	F	151 (58-398)		157 (114-236)
Untreated disease duration (years)	M & F	0.22 (0.06-1.22)		2.44 (1.80-3.22)
Self-cure rate (annual)	M	0.19 (0.09-0.41)		0.23 (0.11-0.48)
	F	0.19 (0.09-0.41)		0.19 (0.09-0.40)
Untreated-TB mortality rate (annual)	M	0.30 (0.12-0.78)		0.47 (0.19-0.96)
	F	0.30 (0.12-0.78)		0.30 (0.12-0.70)

Prevalence (per 100,000*)	M		303 (176-431)	253 (187-334)
	F		149 (85-213)	171 (127-226)
Notification (per 100,000*)	M		102 (91-112)	104 (94-114)
	F		71 (64-78)	70 (64-77)
Prevalence-to-notification ratio	M:F		1.42 (0.91-2.20)	1.00 (1.00-1.00)

M: male; F: female; M:F: male-to-female ratio; CI: confidence interval; CrI: credible interval; Untreated disease duration: inverse of treatment initiation rate
*Modelled as proportion but shown as number per 100,000 population
All potential scale reduction factors, which equal one at convergence, were between 1.000 and 1.002.

SCENARIO 3D

In Scenario 3D, treatment initiation, self-cure and untreated-TB mortality rates were allowed to differ by gender. Modelled gender-specific prevalence and case notification rates were calculated as:

$$prev_g = \frac{inc}{ti_g + sc_g + mu_g}$$

$$not_g = prev_g * ti_g$$

Results in Web Table 21 show that the model could not generate posterior estimates consistent with empirical data on male prevalence in Viet Nam. The model produced posterior estimates that were consistent with empirical data on gender-specific prevalence and case notification rates and M:F ratios in prevalence-to-notification ratios in Malawi. However, posterior estimates for untreated-TB mortality rate among women were over twice those estimated among men, which is inconsistent with available evidence (26).

Web Table 21: Prior and posterior estimates for Scenario 3D

Viet Nam				
Parameter	Gender	Model priors median (95% CrI)	Empirical data estimate (95% CI)	Model posteriors median (95% CrI)
Incidence rate (annual per 100,000*)	M & F	160 (141-182)		178 (161-197)
Untreated disease duration (years)	M	0.09 (0.02-0.39)		2 (1.48-2.66)
	F	0.11 (0.03-0.38)		1.51 (1.01-2.20)
Self-cure rate (annual)	M	0.19 (0.09-0.40)		0.09 (0.05-0.16)
	F	0.19 (0.09-0.41)		0.23 (0.10-0.56)
Untreated-TB mortality rate (annual)	M	0.30 (0.11-0.78)		0.09 (0.05-0.16)
	F	0.29 (0.11-0.78)		1.52 (0.86-2.43)
Prevalence (per 100,000*)	M		351 (262-440)	258 (195-335)
	F		69 (39-99)	73 (50-105)
Notification (per 100,000*)	M		137 (123-151)	129 (117-142)
	F		47 (42-52)	48 (43-54)
Prevalence-to-notification ratio	M:F		1.75 (1.21-2.58)	1.33 (0.82-2.17)
Malawi				
Parameter	Gender	Model priors median (95% CrI)	Empirical data estimate (95% CI)	Model posteriors median (95% CrI)
Incidence rate (annual per 100,000*)	M & F	289 (202-412)		226 (178-295)

Untreated disease duration (years)	M	0.22 (0.07-1.03)		2.42 (1.54-3.60)
	F	0.23 (0.06-1.89)		2.36 (1.58-3.36)
Self-cure rate (annual)	M	0.19 (0.09-0.41)		0.19 (0.09-0.38)
	F	0.19 (0.09-0.41)		0.24 (0.11-0.53)
Untreated-TB mortality rate (annual)	M	0.30 (0.12-0.78)		0.29 (0.13-0.60)
	F	0.30 (0.12-0.78)		0.65 (0.29-1.25)
Prevalence (per 100,000*)	M		303 (176-431)	249 (161-364)
	F		149 (85-213)	169 (115-237)
Notification (per 100,000*)	M		102 (91-112)	103 (93-113)
	F		71 (64-78)	72 (65-79)
Prevalence-to-notification ratio	M:F		1.42 (0.91-2.20)	1.03 (0.59-1.77)

M: male; F: female; M:F: male-to-female ratio; CI: confidence interval; CrI: credible interval; Untreated disease duration: inverse of treatment initiation rate
*Modelled as proportion but shown as number per 100,000 population
All potential scale reduction factors, which equal one at convergence, were between 1.001 and 1.002.

SCENARIO 4

In Scenario 4, incidence, treatment initiation, self-cure and untreated-TB mortality rates were allowed to differ by gender. Modelled gender-specific prevalence and case notification rates were calculated as:

$$prev_g = \frac{inc_g}{ti_g + sc_g + mu_g}$$

$$not_g = prev_g * ti_g$$

Results in Web Table 22 show that the model produced posterior estimates that were consistent with empirical data on gender-specific prevalence and case notification rates and M:F ratios in prevalence-to-notification ratios in both countries. Posterior estimates for transition rates, and therefore prevalence and case notification rate, do not differ substantially from the main model.

Web Table 22: Prior and posterior estimates for Scenario 4

Viet Nam				
Parameter	Gender	Model priors median (95% CrI)	Empirical data estimate (95% CI)	Model posteriors median (95% CrI)
Incidence rate (annual per 100,000*)	M	245 (192-312)		260 (217-317)
	F	58 (27-125)		71 (57-95)
Untreated disease duration (years)	M	0.09 (0.02-0.39)		2.20 (1.65-2.88)
	F	0.11 (0.03-0.37)		1.00 (0.59-1.58)
Self-cure rate (annual)	M	0.19 (0.09-0.41)		0.17 (0.09-0.32)
	F	0.19 (0.09-0.41)		0.19 (0.09-0.39)
Untreated-TB mortality rate (annual)	M	0.29 (0.11-0.78)		0.22 (0.10-0.42)
	F	0.29 (0.11-0.77)		0.27 (0.11-0.68)
Prevalence (per 100,000*)	M		351 (262-440)	304 (233-389)
	F		69 (39-99)	48 (29-74)
Notification (per 100,000*)	M		137 (123-151)	138 (125-153)

	F		47 (42-52)	48 (43-53)
Prevalence-to-notification ratio	M:F		1.75 (1.21-2.58)	2.21 (1.29-3.93)
Malawi				
Parameter	Gender	Model priors median (95% CrI)	Empirical data estimate (95% CI)	Model posteriors median (95% CrI)
Incidence rate (annual per 100,000*)	M	354 (236-533)		296 (216-417)
	F	151 (57-396)		141 (104-214)
Untreated disease duration (years)	M	0.22 (0.07-1.02)		2.78 (1.80-4.06)
	F	0.23 (0.06-1.87)		1.89 (1.18-2.88)
Self-cure rate (annual)	M	0.19 (0.09-0.41)		0.22 (0.10-0.46)
	F	0.19 (0.09-0.41)		0.19 (0.09-0.40)
Untreated-TB mortality rate (annual)	M	0.30 (0.12-0.78)		0.43 (0.18-0.91)
	F	0.30 (0.12-0.77)		0.31 (0.12-0.73)
Prevalence (per 100,000*)	M		303 (176-431)	287 (188-413)
	F		149 (85-213)	135 (85-203)
Notification (per 100,000*)	M		102 (91-112)	103 (93-114)
	F		71 (64-78)	71 (65-78)
Prevalence-to-notification ratio	M:F		1.42 (0.91-2.20)	1.47 (0.80-2.70)

M: male; F: female; M:F: male-to-female ratio; CI: confidence interval; CrI: credible interval; Untreated disease duration: inverse of treatment initiation rate

*Modelled as proportion but shown as number per 100,000 population

All potential scale reduction factors, which equal one at convergence, were between 1.001 and 1.002.

WEB APPENDIX 6: SENSITIVITY ANALYSES – INCIDENCE RATE

We conducted sensitivity analyses to explore the impact of different disease incidence estimates on model results. Our main analyses, described in the main text and above, relied on disease incidence estimates from WHO. We also examined disease incidence estimates from the Institute for Health Metrics and Evaluation (IHME) (27) for each of the scenarios shown in Web Table 7.

IHME disease incidence estimates were calculated from IHME estimates of the number of incident cases (all forms) in men and women ≥ 15 years of age (29). These data were matched to the nearest five-year national population estimate for individuals ≥ 15 years of age from the United Nations World Population Prospect (2, 3). Only overall disease incidence estimates for all forms of TB were available, rather than estimates for smear-positive TB alone.

In Viet Nam, the average annual number of incident TB cases over 2006-07 (the years of the prevalence survey) was estimated as 75,987 (95% CI 58,918-95,913) for men and 52,653 (95% CI 41,102-65,680) for women. The nearest population estimates from 2005 report a male population of 29,742,533 and a female population of 31,595,138. The corresponding disease incidence rate is estimated at 255 (95% CI 198-322) per 100,000 men and 167 (95% CI 130-208) per 100,000 women. These estimates given an overall disease incidence rate of 210 (95% CI 163-263) per 100,000 population.

In Malawi, the average annual number of incident TB cases over 2013-14 (the years of the prevalence survey) was estimated as 15,028 (95% CI 11,148-20,310) for men and 7,386 (95% CI 5,287-9,704) for women. The nearest population estimates from 2015 report a male population of 4,679,650 and a female population of 4,761,703. The corresponding disease incidence rate is estimated at 321 (95% CI 238-434) per 100,000 men and 155 (95% CI 111-204) per 100,000 women. These estimates given an overall disease incidence rate of 237 (95% CI 174-318) per 100,000 population.

Disease incidence estimates were fitted to log-normal distributions with the middle 95% of probabilities falling within the 95% confidence interval, as shown in Web Table 23.

Web Table 23: Data, model estimates and prior distributions for disease incidence (per 100,000) based on IHME estimates

Country	Gender	Data point estimate (95% CI)	Model median (95% CrI)	Distribution in WinBUGS
Viet Nam	M & F	210 (163-263)	207 (163-263)	dlnorm(-6.1799733,67.13776025)
	M	255 (198-322)	253 (198-322)	dlnorm(-5.9815162,64.97934133)
	F	167 (130-208)	164 (130-208)	dlnorm(-6.4103892,69.55906531)
Malawi	M & F	237 (174-318)	235 (174-318)	dlnorm(-6.0523721,42.25977497)
	M	321 (238-434)	321 (238-434)	dlnorm(-5.7402679,42.57298873)
	F	155 (111-204)	151 (111-204)	dlnorm(-6.4991004,41.48650512)

M: male; F: female; CI: confidence interval; CrI: credible interval
*Modelled as proportion but shown as number per 100,000 population

WHO and IHME disease incidence priors are compared below in Web Web Figure ***3*** for Viet Nam and Web Web Figure ***4*** for Malawi.

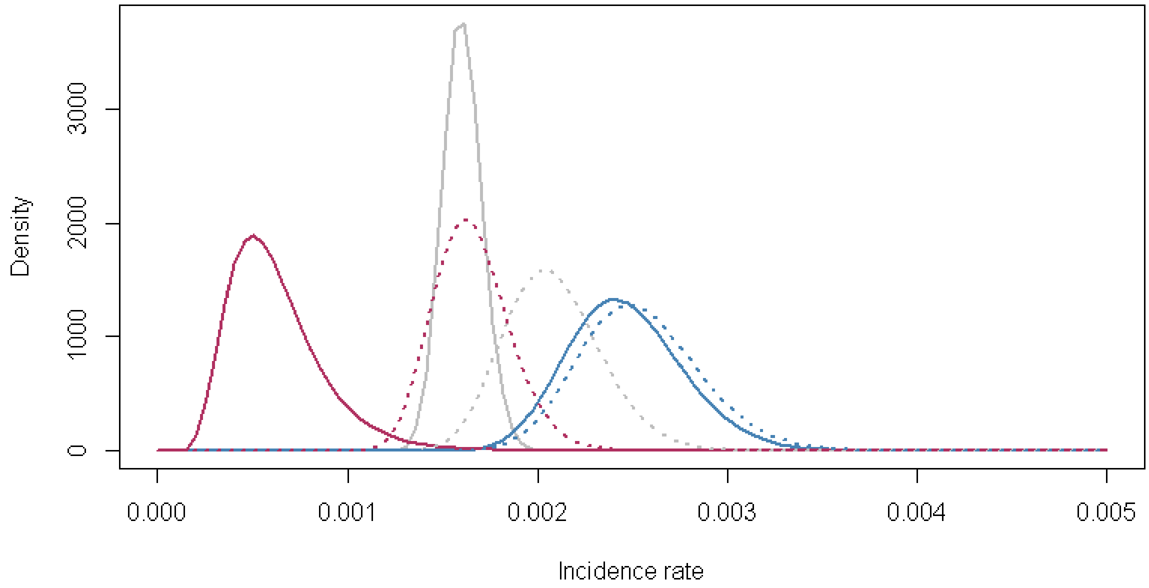

Web Figure 3: Comparison of WHO and IHME disease incidence distributions for Viet Nam
Solid lines: WHO; dashed lines: IHME; grey: overall estimates; light red: female estimates; blue: male estimates

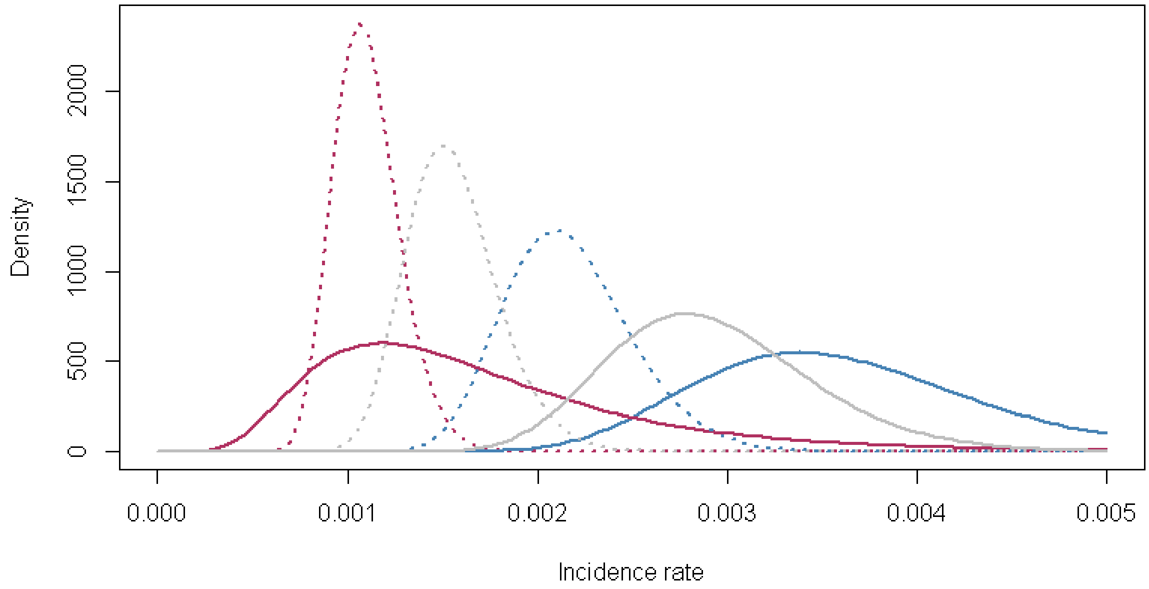

Web Figure 4: Comparison of WHO and IHME incidence distributions for Malawi Solid lines: WHO; dashed lines: IHME; grey: overall estimates; light red: female estimates; blue: male estimates.

Results from the main model using incidence rate priors based on IHME estimates are shown below in Web Table 24. While posterior estimates for Malawi were consistent with empirical data on prevalence, case notification rate and M:F ratios in prevalence-to-notification ratios, posterior estimates for Viet Nam were not consistent with empirical data on male prevalence or M:F ratios in prevalence-to-notification ratios. This is likely a result of IHME underestimating gender disparity in disease incidence in the country, where WHO estimates that incidence is over four times higher in men than in women, while IHME estimates that incidence among men is only 50% higher than among women.

Web Table 24: Model priors, empirical data and model posteriors for sensitivity analyses using IHME estimates for disease incidence priors in Viet Nam and Malawi

Viet Nam				
Parameter	Gender	Model priors median (95% CrI)	Empirical data estimate (95% CI)	Model posteriors median (95% CrI)
Incidence rate (annual per 100,000*)	M	253 (198-322)		327 (273-395)
	F	164 (130-208)		117 (95-144)
Untreated disease duration (years)	M	0.09 (0.02-0.39)		1.89 (1.41-2.50)
	F	0.11 (0.03-0.38)		1.82 (1.26-2.54)
Self-cure rate (annual)	M & F	0.19 (0.09-0.41)		0.23 (0.11-0.48)
Untreated-TB mortality rate (annual)	M & F	0.29 (0.11-0.77)		0.49 (0.23-0.83)
Prevalence (per 100,000*)	M		351 (262-440)	258 (196-333)
	F		69 (39-99)	90 (64-122)
Notification (per 100,000*)	M		137 (123-151)	136 (123-151)
	F		47 (42-52)	50 (44-55)
Prevalence-to-notification ratio	M:F		1.75 (1.21-2.58)	1.04 (0.69-1.60)
Malawi				
Parameter	Gender	Model priors median (95% CrI)	Empirical data estimate (95% CI)	Model posteriors median (95% CrI)
Incidence rate (annual per 100,000*)	M	321 (238-434)		288 (224-370)
	F	151 (111-204)		154 (124-195)
Untreated disease duration (years)	M	0.22 (0.07-1.03)		2.81 (1.89-4.02)
	F	0.23 (0.06-1.86)		1.83 (1.20-2.67)
Self-cure rate (annual)	M & F	0.19 (0.09-0.41)		0.22 (0.10-0.43)
Untreated-TB mortality rate (annual)	M & F	0.30 (0.12-0.78)		0.40 (0.18-0.73)
Prevalence (per 100,000*)	M		303 (176-431)	290 (198-407)
	F		149 (85-213)	130 (87-187)
Notification (per 100,000*)	M		102 (91-112)	103 (94-114)
	F		71 (64-78)	71 (64-78)
Prevalence-to-notification ratio	M:F		1.42 (0.91-2.20)	1.53 (0.92-2.55)

M: male; F: female; M:F: male-to-female ratio; CI: confidence interval; CrI: credible interval; Untreated disease duration: inverse of treatment initiation rate

*Modelled as proportion but shown as number per 100,000 population

All potential scale reduction factors, which equal one at convergence, were between 1.000 and 1.003.

We also examined additional model structure scenarios described in Web Table 7 using disease incidence priors based on estimates from IHME. (Results from additional scenarios are not shown.)

For both countries, scenarios that allowed both disease incidence and treatment initiation rate to differ by gender (Scenarios 3A, 3B, 4), that allowed treatment initiation and untreated-TB mortality rates to differ by gender (Scenario 2E), and that allowed treatment initiation, self-cure and untreated-TB mortality rates to differ by gender (Scenario 3D) were consistent with empirical data on gender-specific prevalence and case notification rates and M:F ratios in prevalence-to-notification ratios. In each of these models – with the exception of Scenario 3A in Malawi – posterior estimates for untreated-TB mortality rate among women showed extreme gender differences that are not considered feasible in light of previous studies showing no evidence of a gender difference in untreated-TB mortality (26). For Scenario 3A in Malawi, posterior estimates in incidence, treatment initiation, self-

cure and untreated-TB mortality rates (and therefore prevalence and case notification rate) did not differ substantially from posterior estimates using the main model.

WEB APPENDIX 7: SENSITIVITY ANALYSES – TREATMENT INITIATION RATE

We conducted sensitivity analyses to explore the implications of the assumption that self-reported symptom duration prior to treatment accurately describes untreated disease duration by setting model priors for untreated disease duration equal to the median self-reported symptom duration prior to treatment for each gender in each country.

Results in Web Table 25 show the model could not generate posterior estimates consistent with empirical data on prevalence in either gender nor case notification rate in men nor M:F ratios in prevalence-to-notification ratios. In both countries, posterior prevalence estimates were substantially lower than estimates from recent prevalence surveys.

In Viet Nam, posterior prevalence estimates were 4% of those reported in survey results for men and 7% those for women. In Malawi, posterior prevalence estimates were 7% of those reported in survey results for men and 12% those for women.

Web Table 25: Model priors, empirical data and model posteriors for sensitivity analyses assuming self-reported symptom duration prior to treatment accurately describes untreated disease duration in Viet Nam and Malawi

Viet Nam				
Parameter	Gender	Model priors median (95% CrI)	Empirical data estimate (95% CI)	Model posteriors median (95% CrI)
Incidence rate (annual per 100,000*)	M	245 (192-312)		176 (160-193)
	F	58 (27-125)		53 (47-60)
Untreated disease duration (years)	M	0.09 (0.09-0.09)		0.09 (0.09-0.09)
	F	0.10 (0.10-0.10)		0.10 (0.10-0.10)
Self-cure rate (annual)	M & F	0.19 (0.09-0.41)		0.20 (0.10-0.44)
Untreated-TB mortality rate (annual)	M & F	0.29 (0.11-0.78)		0.35 (0.12-0.99)
Prevalence (per 100,000*)	M		351 (262-440)	13 (12-15)
	F		69 (39-99)	5 (4-6)
Notification (per 100,000*)	M		137 (123-151)	168 (153-183)
	F		47 (42-52)	50 (45-56)
Prevalence-to-notification ratio	M:F		1.75 (1.21-2.58)	0.80 (0.80-0.80)
Malawi				
Parameter	Gender	Model priors median (95% CrI)	Empirical data estimate (95% CI)	Model posteriors median (95% CrI)
Incidence rate (annual per 100,000*)	M	354 (236-533)		131 (116-164)
	F	151 (57-396)		89 (78-116)
Untreated disease duration (years)	M	0.20 (0.20-0.20)		0.20 (0.20-0.20)
	F	0.24 (0.24-0.24)		0.24 (0.24-0.24)
Self-cure rate (annual)	M & F	0.19 (0.09-0.41)		0.22 (0.10-0.50)
Untreated-TB mortality rate (annual)	M & F	0.30 (0.12-0.77)		0.54 (0.17-2.01)
Prevalence (per 100,000*)	M		303 (176-431)	23 (21-25)
	F		149 (85-213)	18 (16-20)
Notification (per 100,000*)	M		102 (91-112)	113 (103-123)
	F		71 (64-78)	75 (68-82)

Prevalence-to-notification ratio	M:F		1.42 (0.91-2.20)	0.83 (0.83-0.83)

M: male; F: female; M:F: male-to-female ratio; CI: confidence interval; CrI: credible interval; Untreated disease duration: inverse of treatment initiation rate

*Modelled as proportion but shown as number per 100,000 population

All potential scale reduction factors, which equal one at convergence, were between 1.000 and 1.002.

WEB REFERENCES

1. World Health Organization. Tuberculosis country profiles. Geneva, Switzerland: World Health Organization; 2016. (http://www.who.int/tb/country/data/profiles/en/). (Accessed 09 August 2016).

2. United Nations Department of Economic and Social Affairs Population Division. File POP/8-2: Male population by broad age group, major area, region and country, 1950-2100 (thousands). 2015. (https://esa.un.org/unpd/wpp/Download/Standard/Population/). (Accessed 09 August 2016).

3. United Nations Department of Economic and Social Affairs Population Division. File POP/8-3: Female population by broad age group, major area, region and country, 1950-2100 (thousands). 2015. (https://esa.un.org/unpd/wpp/Download/Standard/Population/). (Accessed 09 August 2016).

4. World Health Organization. Global tuberculosis report 2017: Technical appendix. Geneva, Switzerland: World Health Organization, 2017.

5. Hoa NB, Tiemersma EW, Sy DN, et al. Health-seeking behaviour among adults with prolonged cough in Vietnam. *Tropical medicine & international health : TM & IH* 2011;16(10):1260-7.

6. Long NH, Johansson E, Lonnroth K, et al. Longer delays in tuberculosis diagnosis among women in Vietnam. *Int J Tuberc Lung Dis* 1999;3(5):388-93.

7. Thorson A, Hoa N, Long N. Health-seeking behaviour of individuals with a cough of more than 3 weeks. *Lancet* 2000;356(9244):1823-4.

8. Huong NT, Vree M, Duong BD, et al. Delays in the diagnosis and treatment of tuberculosis patients in Vietnam: a cross-sectional study. *BMC Public Health* 2007;7:110.

9. Weiss MG, Auer C, Somma D, et al. Gender and tuberculosis: cross-site analysis and implications of a multi-country study in Bangladesh, India, Malawi, and Colombia. Geneva, Switzerland: World Health Organization, 2006.

10. Gosoniu GD, Ganapathy S, Kemp J, et al. Gender and socio-cultural determinants of delay to diagnosis of TB in Bangladesh, India and Malawi. *Int J Tuberc Lung Dis* 2008;12(7):848-55.

11. Crampin A, Glynn J, Floyd S, et al. Tuberculosis and gender: exploring the patterns in a case control study in Malawi. *Int J Tuberc Lung Dis* 2004;8(2):194-203.

12. Squire S, Belaye A, Kashoti A, et al. 'Lost' smear-positive pulmonary tuberculosis cases: where are they and why did we lose them? *Int J Tuberc Lung Dis* 2005;9(1):25-31.

13. Makwakwa L, Sheu ML, Chiang CY, et al. Patient and health system delays in the diagnosis and treatment of new and retreatment pulmonary tuberculosis cases in Malawi. *BMC Infect Dis* 2014;14:132.

14. Weiss MG, Auer C, Somma D, et al. Gender and tuberculosis: cross-site analysis and implications of a multi-country study in Bandladesh, India, Malawi, and Colombia. Geneva, Switzerland: World Health Organization, 2006.

15. Dye C, Garnett GP, Sleeman K, et al. Prospects for worldwide tuberculosis control under the WHO DOTS strategy. *Lancet* 1998;352(9144):1886–91.

A Bayesian approach to understanding gender differences in tuberculosis disease burden
Katherine C. Horton, Tom Sumner, Rein M. G. J. Houben, Elizabeth L. Corbett, Richard G. White

16. Menzies NA, Cohen T, Lin H-H, et al. Population health impact and cost-effectiveness of tuberculosis diagnosis with Xpert MTB/RIF: a dynamic simulation and economic evaluation. *PLoS Med* 2012;9(11):e1001347.

17. Houben R, Lalli M, Sumner T, et al. TIME Impact–a new user-friendly tuberculosis (TB) model to inform TB policy decisions. *BMC Med* 2016;14:56.

18. World Health Organization. World Health Statistics 2016 - Annex B: Tables of health statistics by country, WHO region and globally. 2016. (http://who.int/entity/gho/publications/world_health_statistics/2016/en/index.html). (Accessed 08 November 2016).

19. Hoa NB, Sy DN, Nhung NV, et al. National survey of tuberculosis prevalence in Viet Nam. *Bull World Health Organ* 2010;88(4):273-80.

20. Hoa NB, Sy DN, Nhung NV, et al. National survey of tuberculosis prevalence in Viet Nam. *Bull World Health Organ* 2010;88(4):273–80.

21. Ministry of Health. Malawi National Prevalence Survey (2013-2014): Technical Report. Lilongwe, Malawi: National TB Control Programme, Ministry of Health, 2016.

22. World Health Organization. Case notifications. Geneva, Switzerland: World Health Organization; 2015. (http://www.who.int/tb/country/data/download/en/). (Accessed 09 August 2016).

23. World Health Organization. Case notifications. Geneva, Switzerland: World Health Organization; 2015. (http://www.who.int/tb/country/data/download/en/). (Accessed).

24. R Core Team. A language and environment for statistical computing. Vienna, Austria: R Foundation for Statistical Computing, 2015.

25. Lunn D, Thomas A, Best N, et al. WinBUGS -- a Bayesian modelling framework: concepts, structure, and extensibility. *Stat Comput* 2000;10:325–37.

26. Tiemersma EW, van der Werf MJ, Borgdorff MW, et al. Natural history of tuberculosis: duration and fatality of untreated pulmonary tuberculosis in HIV negative patients: a systematic review. *PLoS One* 2011;6(4):e17601.

27. Global Burden of Disease Study 2015Collaborative Network. Global Burden of Disease Study 20152016 (GBD 2015) Results2016) Burden by Risk 1990-2016. Seattle, WA: Institute for Heatlh Metrics and Evaluation, 20162017.

28. Global Burden of Disease Study 2015. Global Burden of Disease Study 2015 (GBD 2015) Results. Seattle, WA: Institute for Heatlh Metrics and Evaluation, 2016.

29. Global Burden of Disease Collaborative Network. Global Burden of Disease Study 2016 (GBD 2016) Burden by Risk 1990-2016. Seattle, WA: Institute for Heatlh Metrics and Evaluation, 2017.
